# Estimation of Disability Weights in the General Population of South Korea Using a Paired Comparison

**DOI:** 10.1371/journal.pone.0162478

**Published:** 2016-09-08

**Authors:** Minsu Ock, Jeonghoon Ahn, Seok-Jun Yoon, Min-Woo Jo

**Affiliations:** 1 Department of Preventive Medicine, University of Ulsan, Seoul, South Korea; 2 Department of Health Convergence, Ewha Womans University, Seoul, South Korea; 3 Department of Preventive Medicine, Korea University, Seoul, South Korea; INDEPTH Network, GHANA

## Abstract

We estimated the disability weights in the South Korean population by using a paired comparison-only model wherein ‘full health’ and ‘being dead’ were included as anchor points, without resorting to a cardinal method, such as person trade-off. The study was conducted via 2 types of survey: a household survey involving computer-assisted face-to-face interviews and a web-based survey (similar to that of the GBD 2010 disability weight study). With regard to the valuation methods, paired comparison, visual analogue scale (VAS), and standard gamble (SG) were used in the household survey, whereas paired comparison and population health equivalence (PHE) were used in the web-based survey. Accordingly, we described a total of 258 health states, with ‘full health’ and ‘being dead’ designated as anchor points. In the analysis, 4 models were considered: a paired comparison-only model; hybrid model between paired comparison and PHE; VAS model; and SG model. A total of 2,728 and 3,188 individuals participated in the household and web-based survey, respectively. The Pearson correlation coefficients of the disability weights of health states between the GBD 2010 study and the current models were 0.802 for Model 2, 0.796 for Model 1, 0.681 for Model 3, and 0.574 for Model 4 (all *P-*values<0.001). The discrimination of values according to health state severity was most suitable in Model 1. Based on these results, the paired comparison-only model was selected as the best model for estimating disability weights in South Korea, and for maintaining simplicity in the analysis. Thus, disability weights can be more easily estimated by using paired comparison alone, with ‘full health’ and ‘being dead’ as one of the health states. As noted in our study, we believe that additional evidence regarding the universality of disability weight can be observed by using a simplified methodology of estimating disability weights.

## Introduction

Measuring disease burden is essential in order to set health service and research priorities [1]. However, quantifying disease burden is challenging. Although some epidemiological indicators, such as mortality and morbidity, have been used as measures of disease burden, there is a need for a common single measure reflecting various aspects of a disease [2]. In the global burden of disease (GBD) study in 1990, disability-adjusted life year (DALY) was used as a summary measure that reflects both the mortality and morbidity aspects of diseases [3]. Similar to quality-adjusted life year, DALY combines the impact of mortality and the occurrence and severity of diseases into a single index, thereby enabling disease burden to be compared between different diseases [4].

DALYs are the sum of 2 components: years of life lost (YLLs) and years lived with the disability (YLDs). YLLs reflect premature mortality, whereas YLDs represent the time period living with a disability, i.e., short or long-term loss of health [5]. YLDs can be regarded as the difference in disability between fully healthy people and diseased people, and are calculated by multiplying the number of people with disease or sequela by a relevant disability weight via a prevalence-based approach [6]. The disability weight for a health state quantifies the severity of disease, sequela as a percentage reduction from full health and has value ranging from 0 to 1, with 0 representing full health and 1 indicating being dead. In DALYs, the disability weights act as a bridge between mortality and morbidity.

Several studies have attempted to estimate the disability weights for the GBD or the national burden of disease studies by modifying and adapting methodologies [6–11]. However, the appropriate method to estimate disability weights and validity as well as the universality of the estimated disability weights remain controversial [12–15]. To address these controversies, the disability weights for 220 health states were estimated through an adapted methodology in the recent GBD 2010 disability weight study [6]. In that study, highly consistent results on disability weights were obtained through household surveys in 5 countries and a web-based survey by using a paired comparison and population health equivalence (PHE, a modified form of person trade off). Recently, in the GBD 2013 study, the disability weights were modified from the previous versions by including the results from a European disability weight study [16].

Nevertheless, several aspects of the assessment of disability weights, such as the methodological design and the validity of the values, were criticized following the publication of the GBD 2010 disability weight study [17, 18]. In particular, the universality of the disability weights was questioned, indicating a need for more empirical evidence on universal disability weights and selection of health states [19–21]. Furthermore, considering the disadvantages of using person trade off, such as the lack of theoretical basis and cognitive burden [22], determining an easier way to estimate disability weights is necessary. By adapting the current methodology of estimating disability weights, we believe that empirical evidence on the universality of disability weights can be determined.

In the present study, we estimated disability weights by using a paired comparison-only model wherein ‘full health’ and ‘being dead’ were included as anchor points, without resorting to a cardinal method such as person trade-off. In particular, we calculated and compared the disability weights from 4 different models: a paired comparison-only model, hybrid model between a paired comparison and PHE; visual analogue scale (VAS) model; and standard gamble (SG) model.

## Material and Methods

### Study design and participants

The study was conducted through a household survey and a web-based survey in South Korea, in the same way as in the GBD 2010 disability weight study [6]. The household survey was performed from August 2014 to November 2014 whereas the web-based survey was performed from September 2014 to November 2014. The household survey was conducted using computer-assisted face-to-face interviews, and the web-based survey was available only in the Korean language. This study was approved by the institutional review board of Asan Medical Center (S2014-1396-0002), and written informed consent was obtained from participants prior to household survey participation.

For the household survey, the target population was adults (≥19 years of age) living in South Korea. To select a representative of the Korean population, a total of 2728 representative general samples were drawn from the target population by using a multistage stratified quota method. Sample quotas were predefined considering regions, gender, age, and educational level, as defined by the June 2013 resident registration data, available through the Ministry of Administration and Security, South Korea. The household survey participants were contacted while walking on the street along with quotas and were asked to participate in the survey. Each household survey participant received approximately US$ 9 for completing the survey. For the web-based survey, participants were recruited through advertising in medical colleges and hospitals; announcement at medical meetings and conferences; and word of mouth from other participants involved in the web-based survey.

### Health states

We tested a total of 258 health states, which reflected a diversity of health outcomes as a consequence of disease. Each health state was described by brief lay descriptions that explained the meaning of that health state in terms of several aspects of health [6]. Among the 258 health states, 220 were taken from the GBD 2010 disability weight study and 11 health states were related to environmental diseases as described in the Korea national burden of disease 2012 study. The health states related to environmental diseases were developed by authors (M Ock and MW Jo) based on the existing lay descriptions from the GBD 2010 disability weight study to enhance comparability between health states. We attached a supplemental file with the lay descriptions in English of the added health states (S1 File).

Among the remaining health states, 25 were derived from the EQ-5D-5L health states selected from an orthogonal design [23]. Lastly, two health states (‘full health’ and ‘being dead’) were included as anchor points. M Ock first translated the 220 health states from the GBD 2010 disability weight study into Korean, and MJ Jo revised them. Back translation was performed by a bilingual person and rechecked by M Ock and MJ Jo.

### Survey procedure and interviewer training

In both surveys, participants were initially asked about their gender, age, and educational level. Thereafter, the participants evaluated randomly selected health states by using valuation methods. Different valuation methods were used for the household survey and the web-based survey. Paired comparison, VAS, and SG were used in the household survey, whereas paired comparison and PHE were applied in the web-based survey. Visual aids for SG were used to help participants understand the changes of probability. After the evaluation of health states, participants were additionally asked about other socio-demographic factors, such as current job, income, and clinical information, such as ambulatory care visit in the past 2 weeks, hospitalization in the past 12 months, and morbidities. In the case of morbidities, we asked participants whether they currently had any diseases.

The interviewers of the household survey were explained the survey procedure and health states and were trained to perform each valuation method. All interviewers performed 2 pilot tests before conducting field surveys. The total training time for the interviewers was approximately 2.5 hours.

### Valuation method

The participants in the household survey were asked to elicit their preferences of health states by using 3 valuation methods (paired comparison, VAS, and SG). First, in the paired comparison, the participants were asked to select the healthier option between 2 health states, which were randomly extracted from among the 258 health states (including ‘full health’ and ‘being dead’). Each participant conducted a total of 15 paired comparisons.

Second, in the VAS, the participants were asked to rate the proposed health state on a scale from 0 to 100, with 0 representing the worst imaginable health state and 100 indicating the best imaginable health state. Each participant performed a total of 3 VAS tests. For the first and second VAS tests, 2 health states were randomly selected from among the 256 health states (excluding ‘full health’ and ‘being dead’), while ‘being dead’ was assessed in the third VAS.

Third, in the SG, we asked participants to choose between 2 health states, wherein 1 health state was randomly selected from among the 256 health states (excluding ‘full health’ and ‘being dead’) and the other one was ‘being dead’. Each participant conducted the SG 3 times. If the first health state was regarded as worse than ‘being dead’, the next SG question was asked. If the health state was regarded as better than ‘being dead’, the participants were asked to choose between remaining in that particular health state for rest of their life or having an alternative treatment that could result in the restoration to full health or in immediate death. The questions were continued until the participant did not have a preference between the 2 options. The minimum probability interval of SG was 5%. The probability of 2 choices started at 50% and changed by 5% depending on the participant’s response. Overlap was possible in the health states included in the paired comparison, VAS, and SG.

The participants in the web-based survey were asked to evaluate health states by using 2 valuation methods: paired comparison and PHE. As in the household survey, in the paired comparison, the participants were asked to choose the healthier option between 2 health states, which were randomly extracted from among the 258 health states (including ‘full health’ and ‘being dead’). Each participant performed a total of 15 paired comparisons.

In the PHE, participants were asked to choose the better option between 2 different programs [6]. The first (‘program A’) was a life-saving program, in which 1,000 people were prevented from getting a fatal illness causing rapid death. The second (‘program B’) was a disease-prevention program, in which a certain number of patients with the proposed health state (randomly selected from 1,500, 2,000, 3,000, 5,000, and 10,000) were prevented from getting a less fatal illness. The health state for ‘program B’ was randomly selected from among 256 health states (excluding ‘full health’ and ‘being dead’). If the participant thought ‘program A’ produced a greater overall population health benefit, the number of patients for ‘program B’ increased to the next higher value, from among 1,500, 2,000, 3,000, 5,000, and 10,000. In contrast, if the participant though ‘program B’ produced a greater overall population health benefit, the number of patients for ‘program B’ decreased to the next lower value, from among 1,500, 2,000, 3,000, 5,000, and 10,000. The questions were continued until the choice was altered from ‘program A’ to ‘program B’ or vice versa or until the number of patients for ‘program B’ could no longer be increased or decreased.

### Analysis

Descriptive analyses for the socio-demographic factors were first conducted. Then, the disability weights of the health states for each participant were evaluated. Four models were considered: a paired comparison-only model (Model 1); a hybrid model between paired comparison and PHE (Model 2); a VAS model (Model 3); and a SG model (Model 4).

In Model 1, we randomly selected 80% of the data from the pooled paired comparison data including data from the household survey and the web-based survey. The remaining 20% of the paired comparison data were used to assess the fit of Model 1. Probit regression, which has been commonly used in the analysis of paired comparison data [24], was applied with the stated paired comparison choice as the dependent variable. The 258 health states were regarded as independent variables and treated as dummy variables with ‘being dead’ as the reference. From the coefficient estimates of each health state, we calculated the predicted probabilities. To anchor the transformed predicted probabilities of health states on the disability weight scale ranging from 0 to 1, we used that of ‘being dead (1)’ and ‘full health (0)’ as anchor points. The mean absolute difference was assessed between the observed probability of being selected from the 20% paired comparison data and the predicted probability from the 80% paired comparison data.

In Model 2, we used pooled paired comparison data including those from the household survey and the web-based survey and PHE data from the web-based survey. Initially, we obtained the predicted probabilities from paired comparison data in the same manner as in Model 1, and performed interval regression analysis to obtain the predicted probabilities from PHE data, by adapting the methodology used in the GBD 2010 disability weight study [6]. To link the predicted probabilities between the paired comparison and disability weight estimates derived from the PHE, linear regression was applied with the disability weight estimates from PHE as the dependent variables and the predicted probabilities from the paired comparison as the independent variables. We obtained the predicted probabilities by using the coefficient estimates of each health state and regarded them as disability weights for Model 2.

In Model 3 and Model 4, the concept of disutility was applied; disutility is defined as 1 minus the utility and was assumed to be equal to the disability weight. For Model 3, we used VAS data from the household survey. The utility weights of health were estimated with the formula: ‘VAS values of the health state/100’, if the VAS value of ‘being dead’ was evaluated as 0. In contrast, the utility weights of the health states were estimated with the formula: ‘(VAS values of the health state–VAS values of ‘being dead’)/(100-VAS values of ‘being dead’)’, if the VAS values of ‘being dead’ was not evaluated as 0. Similar to other models, we obtained the predicted probabilities by using linear regression and regarded them as the disability weights for Model 3.

For Model 4, we used SG data from the household survey. The utility weights of the health states were calculated differently according to the response obtained in the comparison between the proposed health state and ‘being dead’. If the health state was evaluated as better than ‘being dead’, the utility weight of the health state was calculated as the possibility of the restoration to full health. On the other hand, the utility weights of the health states that were evaluated as worse than ‘being dead’ were censored at 0 utility weight. As in Model 3, the disutility of each health state was calculated using the formula: ‘1 –utility = disutility’. In addition, we estimated the predicted probabilities by using linear regression and considered them as the disability weights for Model 4.

We calculated the 95% confidence intervals of the disability weights by using the 95% confidence intervals of the predicted probabilities in each model. The frequency distributions of the disability weights from the models were determined and the Pearson correlation coefficients were calculated to compare the disability weights from these models to those obtained in the GBD 2010 disability weight study. Furthermore, the values of ‘1 minus the disability weights’ from the EQ-5D-5L were compared with the utility weights from the EQ-5D-5L, which were derived from the EQ-5D-5L valuation study in Korea, to evaluate the validity of the disability weights in the best model [25]. All statistical analyses were conducted using the Stata 13.1 software (StataCorp, College Station, TX). The Stata code is available from the author upon request. *P*-values below 0.05 were considered statistically significant.

## Results

A total of 2,728 individuals participated in the household survey and 3,188 participated in the web-based survey. The details of the participants’ socio-demographic factors and clinical information for each survey are summarized in Table 1. Those who participated in the web-based survey tended to be younger, have female gender, be involved in non-manual labor, and have higher levels of education and monthly household income, as compared to those who participated in the household survey. However, the people who participated in the household survey tended to have a lower number of clinically relevant medical problems as compared to the participants in the web-based survey.

**Table 1:** Socio-demographic and clinical information for the study participants.

		Household survey		Web-based survey		*P*-value^b^
		N	%	N	%	
Age(years)	19–29	485	17.8	1,960	61.5	< 0.001
	30–39	526	19.3	526	16.5	
	40–49	584	21.4	370	11.6	
	50–59	533	19.5	288	9.0	
	≥60	600	22.0	44	1.4	
Gender	Man	1,360	49.9	1,506	47.2	0.045
	Woman	1,368	50.2	1,682	52.8	
Education level	Elementary school graduate or below	107	3.9	2	0.1	< 0.001
	Middle school graduate	243	8.9	11	0.4	
	High school graduate or attending college	1,741	63.8	1,955	61.3	
	College graduate or above	637	23.4	1,220	38.3	
Occupation	Non-manual	580	21.3	1,161	36.4	< 0.001
	Manual	1,349	49.5	203	6.4	
	Others (including housewife and student)	799	29.3	1,824	57.2	
Monthly household income^a^	<US$ 2,270	500	18.3	439	13.8	< 0.001
	Approximately US$ 2,270–4,550	1,514	55.5	851	26.7	
	>US$ 4,550	714	26.2	1,874	58.8	
	No response	0	0.0	24	0.8	
Ambulatory care visit in the past 2 weeks	Yes	348	12.8	964	30.2	< 0.001
	No	2,380	87.2	2,224	69.8	
Hospitalization in the past 12 month	Yes	79	2.9	248	7.8	< 0.001
	No	2,649	97.1	2,940	92.2	
Morbidity	Yes	331	12.1	576	18.1	< 0.001
	No	2,397	87.9	2,612	81.9	
Total		2,728	100.0	3,188	100.0	-

^**a**^We revised the monthly household income from South Korean won to U.S$ at the exchange rate of 1,100 South Korean won to the US$.

^**b**^From chi-square test

The estimated disability weights for the 256 health states (excluding ‘full health’ and ‘being dead’) from the models and the 220 health states from the GBD 2010 disability weight study are shown in Table 2. The 95% confidence intervals in each model can be found in S1 Table. The frequency distributions of the disability weights of the 220 overlapping health states are presented in Table 3 according to each model. In the GBD 2010 disability weight study, 85.5% of the health states were located below a disability weight of 0.4. However, the frequency distribution of the disability weights from this study differed according to each model. The proportion of health states below a disability weight of 0.4 was 30% in Model 1, 98.6% in Model 2, 22.3% in Model 3, and 85.0% in Model 4. In particular, all health states had a value between 0.2 and 0.5 in Model 2. The disability weights were distributed most evenly in Model 1.

**Table 2:** Disability weights for the health states of each model.

Health states	GBD 2010	Model 1	Model 2	Model 3	Model 4
Infectious disease: acute episode, mild	0.005	0.111	0.232	0.221	0.208
Infectious disease: acute episode, moderate	0.053	0.385	0.304	0.410	0.239
Infectious disease: acute episode, severe	0.210	0.587	0.339	0.511	0.291
Infectious disease: post-acute consequences (fatigue, emotional lability, insomnia)	0.254	0.428	0.317	0.442	0.304
Diarrhoea: mild	0.061	0.359	0.299	0.438	0.300
Diarrhoea: moderate	0.202	0.535	0.320	0.455	0.278
Diarrhoea: severe	0.281	0.614	0.348	0.485	0.366
Epididymo-orchitis	0.097	0.653	0.348	0.641	0.355
Herpes zoster	0.061	0.323	0.284	0.446	0.110
HIV cases: symptomatic, pre-AIDS	0.221	0.337	0.303	0.410	0.213
HIV/AIDS cases: receiving antiretroviral treatment	0.053	0.261	0.284	0.436	0.208
AIDS cases: not receiving antiretroviral treatment	0.547	0.526	0.334	0.478	0.221
Intestinal nematode infections: symptomatic	0.030	0.521	0.331	0.529	0.338
Lymphatic filariasis: symptomatic	0.110	0.473	0.316	0.458	0.234
Ear pain	0.018	0.241	0.273	0.353	0.202
Tuberculosis: without HIV infection	0.331	0.537	0.333	0.422	0.255
Tuberculosis: with HIV infection	0.399	0.461	0.319	0.414	0.361
Cancer: diagnosis and primary therapy	0.294	0.536	0.334	0.521	0.318
Cancer: metastatic	0.484	0.596	0.346	0.607	0.242
Mastectomy	0.038	0.468	0.321	0.450	0.274
Stoma	0.086	0.634	0.359	0.668	0.414
Terminal phase: with medication (for cancers, end-stage kidney/liver disease)	0.508	0.733	0.376	0.637	0.443
Terminal phase, without medication (for cancers, end-stage kidney or liver disease)	0.519	0.737	0.370	0.648	0.461
Acute myocardial infarction: days 1–2	0.422	0.575	0.338	0.553	0.231
Acute myocardial infarction: days 3–28	0.056	0.390	0.303	0.430	0.250
Angina pectoris: mild	0.037	0.252	0.272	0.286	0.153
Angina pectoris: moderate	0.066	0.344	0.287	0.344	0.296
Angina pectoris: severe	0.167	0.470	0.320	0.490	0.340
Cardiac conduction disorders and cardiac dysrhythmias	0.145	0.670	0.356	0.586	0.423
Claudication	0.016	0.320	0.287	0.399	0.304
Heart failure: mild	0.037	0.305	0.281	0.340	0.156
Heart failure: moderate	0.070	0.376	0.292	0.420	0.263
Heart failure: severe	0.186	0.547	0.328	0.489	0.291
Stroke: long-term consequences, mild	0.021	0.209	0.269	0.404	0.206
Stroke: long-term consequences, moderate	0.076	0.270	0.283	0.403	0.323
Stroke: long-term consequences, moderate plus cognition problems	0.312	0.497	0.331	0.517	0.313
Stroke: long-term consequences, severe	0.539	0.768	0.377	0.700	0.396
Stroke: long-term consequences, severe plus cognition problems	0.567	0.809	0.391	0.686	0.559
Diabetic foot	0.023	0.222	0.263	0.315	0.158
Diabetic neuropathy	0.099	0.628	0.347	0.452	0.376
Chronic kidney disease (stage IV)	0.105	0.345	0.299	0.435	0.177
End-stage renal disease: with kidney transplant	0.027	0.200	0.255	0.361	0.122
End-stage renal disease: on dialysis	0.573	0.713	0.365	0.583	0.307
Decompensated cirrhosis of the liver	0.194	0.375	0.303	0.468	0.269
Gastric bleeding	0.323	0.782	0.376	0.567	0.398
Crohn's disease or ulcerative colitis	0.225	0.620	0.348	0.519	0.342
Benign prostatic hypertrophy: symptomatic cases	0.070	0.372	0.296	0.388	0.156
Urinary incontinence	0.142	0.582	0.342	0.627	0.300
Impotence	0.019	0.450	0.309	0.509	0.378
Infertility: primary	0.011	0.325	0.292	0.387	0.168
Infertility: secondary	0.006	0.168	0.250	0.254	0.178
Asthma: controlled	0.009	0.148	0.251	0.239	0.156
Asthma: partially controlled	0.027	0.294	0.283	0.320	0.319
Asthma: uncontrolled	0.132	0.342	0.296	0.412	0.300
COPD and other chronic respiratory problems: mild	0.015	0.173	0.256	0.278	0.144
COPD and other chronic respiratory problems: moderate	0.192	0.439	0.319	0.491	0.339
COPD and other chronic respiratory problems: severe	0.383	0.551	0.326	0.472	0.200
Dementia: mild	0.082	0.401	0.306	0.391	0.242
Dementia: moderate	0.346	0.606	0.345	0.520	0.444
Dementia: severe	0.438	0.804	0.385	0.715	0.463
Headache: migraine	0.433	0.635	0.350	0.513	0.358
Headache: tension-type	0.04	0.452	0.313	0.473	0.303
Multiple sclerosis: mild	0.198	0.428	0.314	0.457	0.402
Multiple sclerosis: moderate	0.445	0.736	0.372	0.588	0.392
Multiple sclerosis: severe	0.707	0.801	0.379	0.661	0.377
Epilepsy: treated, seizure free	0.072	0.449	0.315	0.423	0.450
Epilepsy: treated, with recent seizures	0.319	0.624	0.347	0.527	0.436
Epilepsy: untreated	0.420	0.660	0.356	0.577	0.277
Epilepsy: severe	0.657	0.816	0.386	0.710	0.442
Parkinson's disease: mild	0.011	0.222	0.267	0.423	0.256
Parkinson's disease: moderate	0.263	0.474	0.325	0.491	0.335
Parkinson's disease: severe	0.549	0.742	0.369	0.561	0.528
Alcohol use disorder: mild	0.259	0.463	0.321	0.467	0.294
Alcohol use disorder: moderate	0.388	0.612	0.341	0.489	0.304
Alcohol use disorder: severe	0.549	0.797	0.389	0.562	0.382
Fetal alcohol syndrome: mild	0.017	0.353	0.300	0.402	0.239
Fetal alcohol syndrome: moderate	0.057	0.459	0.314	0.527	0.266
Fetal alcohol syndrome: severe	0.177	0.712	0.367	0.622	0.394
Cannabis dependence	0.329	0.769	0.376	0.587	0.304
Amphetamine dependence	0.353	0.808	0.382	0.569	0.370
Cocaine dependence	0.376	0.738	0.375	0.595	0.356
Heroin and other opioid dependence	0.641	0.814	0.391	0.619	0.550
Anxiety disorders: mild	0.030	0.257	0.278	0.385	0.313
Anxiety disorders: moderate	0.149	0.566	0.333	0.409	0.259
Anxiety disorders: severe	0.523	0.787	0.370	0.551	0.536
Major depressive disorder: mild episode	0.159	0.551	0.333	0.486	0.340
Major depressive disorder: moderate episode	0.406	0.756	0.376	0.530	0.361
Major depressive disorder: severe episode	0.655	0.838	0.391	0.672	0.563
Bipolar disorder: manic episode	0.480	0.658	0.362	0.493	0.326
Bipolar disorder: residual state	0.035	0.248	0.282	0.387	0.225
Schizophrenia: acute state	0.756	0.836	0.388	0.584	0.483
Schizophrenia, residual state	0.576	0.742	0.377	0.548	0.294
Anorexia nervosa	0.223	0.448	0.315	0.373	0.278
Bulimia nervosa	0.223	0.532	0.328	0.477	0.270
Attention deficit hyperactivity disorder	0.049	0.470	0.309	0.373	0.381
Conduct disorder	0.236	0.625	0.339	0.408	0.215
Asperger's syndrome	0.110	0.432	0.317	0.444	0.406
Autism	0.259	0.677	0.357	0.572	0.256
Intellectual disability: mild	0.031	0.493	0.331	0.536	0.347
Intellectual disability: moderate	0.080	0.585	0.340	0.578	0.355
Intellectual disability: severe	0.126	0.652	0.357	0.513	0.406
Intellectual disability: profound	0.157	0.650	0.350	0.560	0.367
Hearing loss: mild	0.005	0.138	0.243	0.283	0.243
Hearing loss: moderate	0.023	0.231	0.279	0.408	0.261
Hearing loss: severe	0.032	0.406	0.308	0.502	0.142
Hearing loss: profound	0.031	0.491	0.325	0.520	0.370
Hearing loss: complete	0.033	0.669	0.350	0.701	0.398
Hearing loss: mild, with ringing	0.038	0.423	0.310	0.397	0.144
Hearing loss: moderate, with ringing	0.058	0.438	0.317	0.525	0.267
Hearing loss: severe, with ringing	0.065	0.449	0.321	0.560	0.252
Hearing loss: profound, with ringing	0.088	0.640	0.346	0.525	0.342
Hearing loss: complete, with ringing	0.092	0.629	0.348	0.503	0.300
Distance vision: mild impairment	0.004	0.084	0.234	0.238	0.150
Distance vision: moderate impairment	0.033	0.475	0.315	0.445	0.367
Distance vision: severe impairment	0.191	0.687	0.364	0.609	0.358
Distance vision blindness	0.195	0.708	0.371	0.647	0.464
Near vision impairment	0.013	0.256	0.269	0.467	0.217
Low back pain: acute, without leg pain	0.269	0.588	0.344	0.474	0.224
Low back pain: acute, with leg pain	0.322	0.595	0.346	0.511	0.319
Low back pain: chronic, without leg pain	0.366	0.524	0.328	0.492	0.354
Low back pain: chronic, with leg pain	0.374	0.554	0.337	0.497	0.273
Neck pain: acute, mild	0.040	0.338	0.294	0.383	0.146
Neck pain: acute, severe	0.221	0.492	0.329	0.491	0.333
Neck pain: chronic, mild	0.101	0.407	0.310	0.387	0.207
Neck pain: chronic, severe	0.286	0.495	0.326	0.390	0.253
Musculoskeletal problems: legs, mild	0.023	0.205	0.268	0.433	0.232
Musculoskeletal problems: legs, moderate	0.079	0.491	0.314	0.459	0.255
Musculoskeletal problems: legs, severe	0.171	0.535	0.337	0.535	0.336
Musculoskeletal problems: arms, mild	0.024	0.344	0.289	0.346	0.271
Musculoskeletal problems: arms, moderate	0.114	0.461	0.320	0.446	0.265
Musculoskeletal problems: generalised, moderate	0.292	0.568	0.339	0.463	0.272
Musculoskeletal problems: generalised, severe	0.606	0.746	0.378	0.554	0.179
Gout: acute	0.293	0.672	0.355	0.476	0.325
Amputation of finger(s), excluding thumb: long term, with treatment	0.030	0.274	0.292	0.382	0.265
Amputation of thumb: long term	0.013	0.272	0.281	0.356	0.129
Amputation of one arm: long term, with or without treatment	0.130	0.634	0.343	0.490	0.389
Amputation of both arms: long term, with treatment	0.044	0.470	0.321	0.462	0.245
Amputation of both arms: long term, without treatment	0.359	0.748	0.367	0.576	0.459
Amputation of toe	0.008	0.252	0.279	0.389	0.213
Amputation of one leg: long term, with treatment	0.021	0.409	0.313	0.477	0.150
Amputation of one leg: long term, without treatment	0.164	0.629	0.345	0.505	0.393
Amputation of both legs: long term, with treatment	0.051	0.492	0.321	0.451	0.387
Amputation of both legs: long term, without treatment	0.494	0.730	0.372	0.613	0.469
Burns of <20% total surface area without lower airway burns: short term, with or without treatment	0.096	0.514	0.320	0.474	0.337
Burns of <20% total surface area or <10% total surface area if head or neck, or hands or wrist involved: long term, with or without treatment	0.018	0.236	0.279	0.387	0.191
Burns of ≥20% total surface area: short term, with or without treatment	0.333	0.514	0.328	0.518	0.335
Burns of ≥20% total surface area or ≥10% total surface area if head or neck, or hands or wrist involved: long term, with treatment	0.127	0.555	0.338	0.489	0.433
Burns of ≥20% total surface area or ≥10% total surface area if head or neck, or hands or wrist involved: long term, without treatment	0.438	0.745	0.372	0.577	0.448
Lower airway burns: with or without treatment	0.373	0.827	0.392	0.588	0.341
Crush injury: short or long term, with or without treatment	0.145	0.325	0.299	0.418	0.276
Dislocation of hip: long term, with or without treatment	0.017	0.311	0.284	0.380	0.100
Dislocation of knee: long term, with or without treatment	0.129	0.598	0.342	0.489	0.328
Dislocation of shoulder: long term, with or without treatment	0.08	0.393	0.308	0.441	0.283
Other injuries of muscle and tendon (includes sprains, strains and dislocations other than shoulder, knee, or hip)	0.009	0.206	0.264	0.286	0.265
Drowning and non-fatal submersion: short or long term, with or without treatment	0.288	0.589	0.332	0.490	0.257
Fracture of clavicle, scapula, or humerus: short or long term, with or without treatment	0.053	0.416	0.314	0.482	0.274
Fracture of face bone: short or long term, with or without treatment	0.173	0.580	0.331	0.392	0.263
Fracture of foot bones: short term, with or without treatment	0.033	0.383	0.293	0.368	0.242
Fracture of foot bones: long term, without treatment	0.033	0.235	0.279	0.387	0.350
Fracture of hand: short term, with or without treatment	0.025	0.238	0.269	0.380	0.173
Fracture of hand: long term, without treatment	0.016	0.117	0.247	0.349	0.219
Fracture of neck of femur: short term, with or without treatment	0.308	0.701	0.358	0.609	0.257
Fracture of neck of femur: long term, with treatment	0.072	0.380	0.301	0.385	0.436
Fracture of neck of femur: long term, without treatment	0.388	0.793	0.383	0.612	0.502
Fracture, other than neck of femur: short term, with or without treatment	0.192	0.702	0.357	0.540	0.380
Fracture, other than neck of femur: long term, without treatment	0.053	0.363	0.303	0.472	0.260
Fracture of patella, tibia or fibula, or ankle: short term, with or without treatment	0.087	0.519	0.322	0.446	0.271
Fracture of patella, tibia or fibula, or ankle: long term, with or without treatment	0.070	0.428	0.308	0.397	0.250
Fracture of pelvis: short term	0.390	0.759	0.370	0.558	0.319
Fracture of pelvis: long term	0.194	0.573	0.338	0.562	0.261
Fracture of radius or ulna: short term, with or without treatment	0.065	0.421	0.310	0.434	0.281
Fracture of radius or ulna: long term, without treatment	0.050	0.473	0.310	0.416	0.193
Fracture of skull: short or long term, with or without treatment	0.073	0.390	0.305	0.425	0.276
Fracture of sternum or fracture of one or two ribs: short term, with or without treatment	0.150	0.500	0.330	0.485	0.353
Fracture of vertebral column: short or long term, with or without treatment	0.132	0.399	0.312	0.495	0.252
Fractures: treated, long term	0.003	0.161	0.247	0.360	0.235
Injured nerves: short term	0.065	0.463	0.321	0.515	0.329
Injured nerves: long term	0.136	0.511	0.317	0.442	0.379
Injury to eyes: short term	0.079	0.409	0.310	0.459	0.215
Severe traumatic brain injury: short term, with or without treatment	0.235	0.479	0.325	0.422	0.267
Traumatic brain injury: long-term consequences, minor, with or without treatment	0.106	0.487	0.314	0.425	0.293
Traumatic brain injury: long-term consequences, moderate, with or without treatment	0.224	0.497	0.332	0.521	0.284
Traumatic brain injury: long-term consequences, severe, with or without treatment	0.625	0.829	0.392	0.592	0.544
Open wound: short term, with or without treatment	0.005	0.208	0.268	0.305	0.270
Poisoning: short term, with or without treatment	0.171	0.400	0.301	0.394	0.241
Severe chest injury: long term, with or without treatment	0.056	0.496	0.315	0.406	0.155
Severe chest injury: short term, with or without treatment	0.352	0.590	0.344	0.453	0.222
Spinal cord lesion below neck: treated	0.047	0.697	0.354	0.575	0.374
Spinal cord lesion below neck: untreated	0.440	0.855	0.401	0.764	0.398
Spinal cord lesion at neck: treated	0.369	0.813	0.391	0.808	0.676
Spinal cord lesion at neck level: untreated	0.673	0.912	0.421	0.696	0.575
Abdominopelvic problem: mild	0.012	0.154	0.242	0.294	0.252
Abdominopelvic problem: moderate	0.123	0.416	0.313	0.492	0.143
Abdominopelvic problem: severe	0.326	0.793	0.383	0.581	0.422
Anaemia: mild	0.005	0.123	0.236	0.250	0.119
Anaemia: moderate	0.058	0.321	0.295	0.374	0.227
Anaemia: severe	0.164	0.453	0.325	0.418	0.300
Periodontitis	0.008	0.095	0.240	0.213	0.353
Dental caries: symptomatic	0.012	0.181	0.260	0.263	0.142
Severe tooth loss	0.072	0.478	0.320	0.523	0.348
Disfigurement: level 1	0.013	0.358	0.301	0.489	0.320
Disfigurement: level 2	0.072	0.605	0.343	0.515	0.374
Disfigurement: level 3	0.398	0.745	0.378	0.569	0.507
Disfigurement: level 1 with itch or pain	0.029	0.419	0.310	0.394	0.281
Disfigurement: level 2, with itch or pain	0.187	0.613	0.345	0.495	0.361
Disfigurement: level 3, with itch or pain	0.562	0.849	0.403	0.682	0.425
Generic uncomplicated disease: worry and daily medication	0.031	0.299	0.284	0.433	0.154
Generic uncomplicated disease: anxiety about diagnosis	0.054	0.411	0.306	0.407	0.369
Iodine-deficiency goiter	0.200	0.548	0.332	0.497	0.302
Kwashiorkor	0.055	0.346	0.291	0.410	0.204
Severe wasting	0.127	0.355	0.291	0.439	0.353
Speech problems	0.054	0.518	0.320	0.447	0.196
Motor impairment: mild	0.012	0.164	0.258	0.344	0.165
Motor impairment: moderate	0.076	0.322	0.295	0.416	0.183
Motor impairment: severe	0.377	0.723	0.367	0.622	0.404
Motor plus cognitive impairments: mild	0.054	0.435	0.312	0.421	0.292
Motor plus cognitive impairments: moderate	0.221	0.509	0.326	0.519	0.294
Motor plus cognitive impairments: severe	0.425	0.790	0.378	0.623	0.444
Rectovaginal fistula	0.492	0.782	0.375	0.586	0.386
Vesicovaginal fistula	0.338	0.686	0.364	0.685	0.350
Allergic rhinitis & conjunctivitis: mild		0.202	0.265	0.295	0.238
Allergic rhinitis & conjunctivitis: severe		0.343	0.293	0.398	0.205
Post-traumatic stress disorder		0.616	0.346	0.430	0.300
Multiple chemical sensitivity		0.486	0.316	0.459	0.308
Tinnitus		0.275	0.277	0.429	0.238
Annoyance: mild		0.169	0.253	0.273	0.278
Annoyance: severe		0.249	0.269	0.326	0.205
Sleep disorder: mild		0.149	0.248	0.280	0.243
Sleep disorder: severe		0.328	0.287	0.428	0.273
Learning disorder: mild		0.157	0.239	0.309	0.152
Learning disorder: severe		0.156	0.254	0.384	0.210
EQ-5D 11111		0.116	0.238	0.162	0.096
EQ-5D 12322		0.404	0.300	0.410	0.175
EQ-5D 22431		0.355	0.304	0.463	0.306
EQ-5D 21225		0.619	0.339	0.521	0.278
EQ-5D 23142		0.514	0.323	0.515	0.231
EQ-5D 52213		0.562	0.334	0.515	0.333
EQ-5D 33251		0.458	0.318	0.480	0.235
EQ-5D 35123		0.576	0.332	0.475	0.270
EQ-5D 34412		0.516	0.331	0.534	0.320
EQ-5D 31334		0.643	0.344	0.477	0.225
EQ-5D 14244		0.673	0.350	0.560	0.306
EQ-5D 13533		0.403	0.313	0.501	0.326
EQ-5D 41443		0.591	0.343	0.563	0.344
EQ-5D 44521		0.615	0.346	0.560	0.271
EQ-5D 42154		0.683	0.362	0.534	0.409
EQ-5D 45232		0.641	0.353	0.532	0.263
EQ-5D 43315		0.670	0.349	0.593	0.279
EQ-5D 24353		0.609	0.341	0.535	0.320
EQ-5D 25514		0.718	0.364	0.549	0.360
EQ-5D 54135		0.719	0.362	0.566	0.309
EQ-5D 53424		0.765	0.380	0.621	0.297
EQ-5D 55341		0.558	0.346	0.505	0.434
EQ-5D 51552		0.744	0.369	0.633	0.315
EQ-5D 32545		0.708	0.359	0.489	0.362
EQ-5D 15455		0.772	0.375	0.580	0.367

Model 1: paired comparison model; Model 2: hybrid model; Model 3: visual analogue scale model; Model 4: standard gamble model

**Table 3:** Distribution of disability weights for the 220 health states from the GBD 2010 study according to each model.

Disability weight	GBD 2010		Model 1		Model 2		Model 3		Model 4		
	N	%	N	%	N	%	N	%	N	%
0.0–0.1	101	45.9	0	0.0	0	0.0	0	0.0	0	0.0
0.1–0.2	38	17.3	13	5.9	0	0.0	0	0.0	30	13.6
0.2–0.3	23	10.5	21	9.6	55	25.0	12	5.5	80	36.4
0.3–0.4	26	11.8	28	12.7	162	73.6	37	16.8	77	35.0
0.4–0.5	13	5.9	40	18.2	3	1.4	87	39.5	23	10.5
0.5–0.6	11	5.0	38	17.3	0	0.0	57	25.9	9	4.1
0.6–0.7	6	2.7	31	14.1	0	0.0	21	9.5	1	0.5
0.7–0.8	2	0.9	23	10.5	0	0.0	5	2.3	0	0.0
0.8–0.9	0	0.0	25	11.4	0	0.0	1	0.5	0	0.0
0.9–1.0	0	0.0	1	0.5	0	0.0	0	0.0	0	0.0

Model 1: paired comparison model; Model 2: hybrid model; Model 3: visual analogue scale model; Model 4: standard gamble model

**Fig 1:**
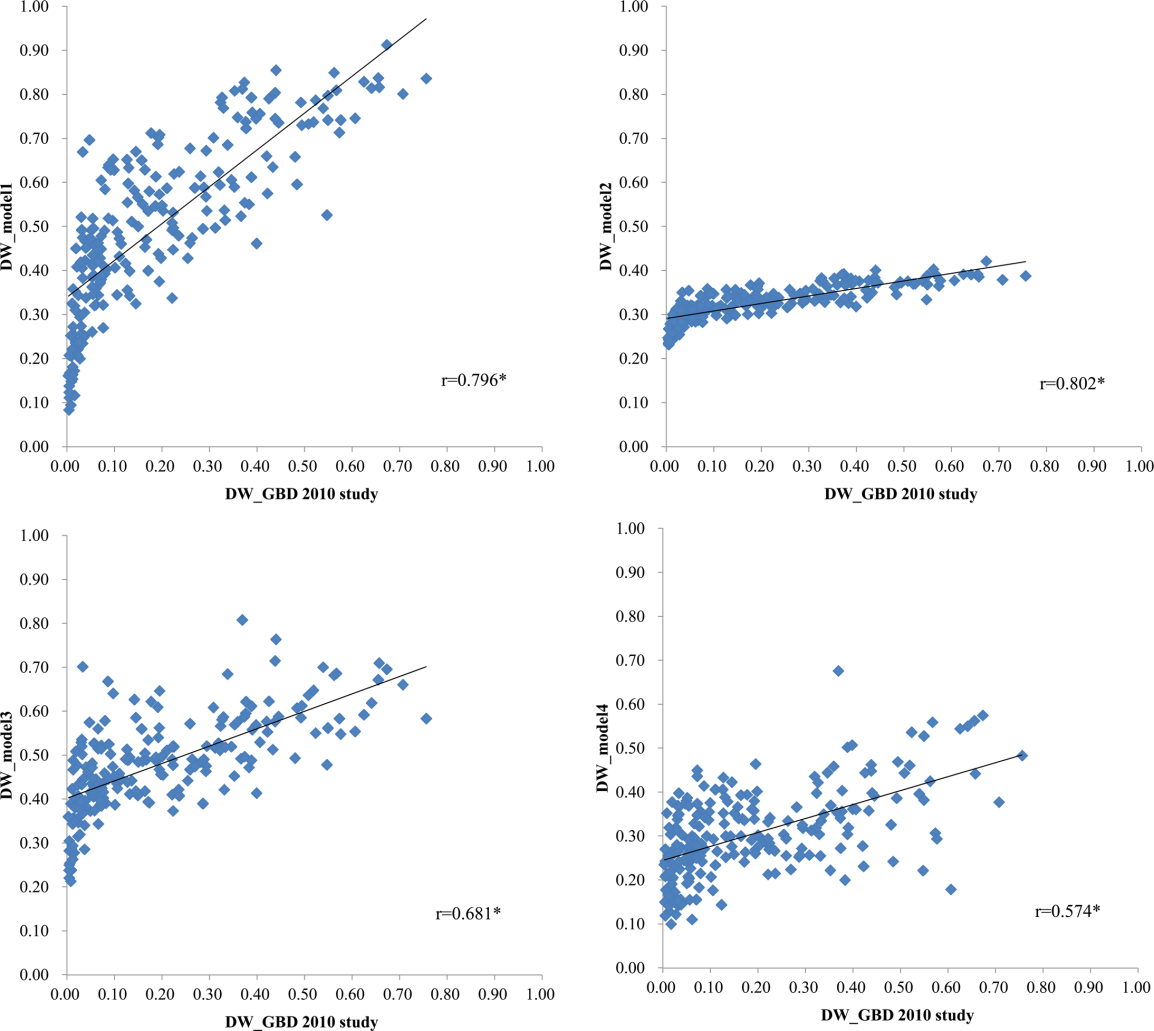
Correlation between the disability weights from the GBD 2010 disability weight study and those from the 4 models used in the present study. *P-value<0.001.

The Pearson correlation coefficients between the disability weights of health states for the GBD 2010 disability weight study and those of the health states for the current models are shown in Fig 1. The highest Pearson correlation coefficient was 0.802 in Model 2, followed by 0.796 in Model 1, 0.681 in Model 3, and 0.574 in Model 4. All Pearson correlation coefficients were statistically significant.

Based on the distribution of disability weights of the health states for each model and the Pearson correlation coefficients, the paired comparison-only model based on probit regression was selected as the best model for estimating the disability weight in South Korea and for maintaining the simplicity of the analysis. The estimated disability weights and 95% confidence intervals for the 256 health states (excluding ‘full health’ and ‘being dead’) from Model 1 are shown in Table 4. The health state with the highest disability weight (0.912) was ‘Spinal cord lesion at neck level: untreated’ (N191 in Table 4), followed by ‘Spinal cord lesion below neck: untreated’ (N189) with a disability weight of 0.691 and ‘Disfigurement: level 3, with itch or pain’ (N206) with a disability weight of 0.849. Furthermore, the disability weights of drug addiction were high as compared to other health states. For example, the disability weights for ‘Heroin and other opioid dependence’ (N82), ‘Amphetamine dependence’ (N80), and ‘Cannabis dependence (N79)’ were 0.814, 0808, and 0.769, respectively. The health state with the lowest disability weight was ‘Distance vision: mild impairment’ (N113) with 0.084, followed by ‘Periodontitis’ (N198) with 0.095, and ‘Infectious diseases: acute episode, mild’ (N1) with 0.111.

**Table 4:** Confidence intervals of the disability weights from the paired comparison-only model.

N	Health states	DW	95% confidence interval
1	Infectious disease: acute episode, mild	0.111	0.061~0.186
2	Infectious disease: acute episode, moderate	0.385	0.294~0.488
3	Infectious disease: acute episode, severe	0.587	0.488~0.681
4	Infectious disease: post-acute consequences (fatigue, emotional lability, insomnia)	0.428	0.336~0.525
5	Diarrhoea: mild	0.359	0.271~0.462
6	Diarrhoea: moderate	0.535	0.438~0.630
7	Diarrhoea: severe	0.614	0.515~0.713
8	Epididymo-orchitis	0.653	0.561~0.737
9	Herpes zoster	0.323	0.240~0.415
10	HIV cases: symptomatic, pre-AIDS	0.337	0.255~0.429
11	HIV/AIDS cases: receiving antiretroviral treatment	0.261	0.187~0.348
12	AIDS cases: not receiving antiretroviral treatment	0.526	0.431~0.621
13	Intestinal nematode infections: symptomatic	0.521	0.424~0.618
14	Lymphatic filariasis: symptomatic	0.473	0.382~0.565
15	Ear pain	0.241	0.169~0.327
16	Tuberculosis: without HIV infection	0.537	0.439~0.652
17	Tuberculosis: with HIV infection	0.461	0.366~0.560
18	Cancer: diagnosis and primary therapy	0.536	0.441~0.629
19	Cancer: metastatic	0.596	0.495~0.691
20	Mastectomy	0.468	0.374~0.568
21	Stoma	0.634	0.542~0.721
22	Terminal phase: with medication (for cancers, end-stage kidney/liver disease)	0.733	0.642~0.811
23	Terminal phase, without medication (for cancers, end-stage kidney or liver disease)	0.737	0.649~0.813
24	Acute myocardial infarction: days 1–2	0.575	0.480~0.682
25	Acute myocardial infarction: days 3–28	0.390	0.300~0.486
26	Angina pectoris: mild	0.252	0.177~0.340
27	Angina pectoris: moderate	0.344	0.258~0.439
28	Angina pectoris: severe	0.470	0.376~0.566
29	Cardiac conduction disorders and cardiac dysrhythmias	0.670	0.555~0.771
30	Claudication	0.320	0.236~0.413
31	Heart failure: mild	0.305	0.226~0.394
32	Heart failure: moderate	0.376	0.286~0.472
33	Heart failure: severe	0.547	0.452~0.639
34	Stroke: long-term consequences, mild	0.209	0.140~0.294
35	Stroke: long-term consequences, moderate	0.270	0.194~0.358
36	Stroke: long-term consequences, moderate plus cognition problems	0.497	0.403~0.591
37	Stroke: long-term consequences, severe	0.768	0.681~0.841
38	Stroke: long-term consequences, severe plus cognition problems	0.809	0.728~0.874
39	Diabetic foot	0.222	0.154~0.308
40	Diabetic neuropathy	0.628	0.531~0.719
41	Chronic kidney disease (stage IV)	0.345	0.257~0.460
42	End-stage renal disease: with kidney transplant	0.200	0.135~0.279
43	End-stage renal disease: on dialysis	0.713	0.626~0.816
44	Decompensated cirrhosis of the liver	0.375	0.285~0.473
45	Gastric bleeding	0.782	0.699~0.850
46	Crohn's disease or ulcerative colitis	0.620	0.526~0.707
47	Benign prostatic hypertrophy: symptomatic cases	0.372	0.284~0.467
48	Urinary incontinence	0.582	0.487~0.672
49	Impotence	0.450	0.358~0.546
50	Infertility: primary	0.325	0.241~0.442
51	Infertility: secondary	0.168	0.109~0.243
52	Asthma: controlled	0.148	0.094~0.227
53	Asthma: partially controlled	0.294	0.215~0.385
54	Asthma: uncontrolled	0.342	0.258~0.445
55	COPD and other chronic respiratory problems: mild	0.173	0.111~0.263
56	COPD and other chronic respiratory problems: moderate	0.439	0.347~0.533
57	COPD and other chronic respiratory problems: severe	0.551	0.455~0.643
58	Dementia: mild	0.401	0.310~0.503
59	Dementia: moderate	0.606	0.513~0.694
60	Dementia: severe	0.804	0.723~0.868
61	Headache: migraine	0.635	0.544~0.725
62	Headache: tension-type	0.452	0.359~0.569
63	Multiple sclerosis: mild	0.428	0.335~0.527
64	Multiple sclerosis: moderate	0.736	0.647~0.812
65	Multiple sclerosis: severe	0.801	0.720~0.872
66	Epilepsy: treated, seizure free	0.449	0.355~0.586
67	Epilepsy: treated, with recent seizures	0.624	0.526~0.714
68	Epilepsy: untreated	0.660	0.566~0.750
69	Epilepsy: severe	0.816	0.740~0.877
70	Parkinson's disease: mild	0.222	0.150~0.308
71	Parkinson's disease: moderate	0.474	0.378~0.573
72	Parkinson's disease: severe	0.742	0.655~0.816
73	Alcohol use disorder: mild	0.463	0.368~0.566
74	Alcohol use disorder: moderate	0.612	0.515~0.702
75	Alcohol use disorder: severe	0.797	0.715~0.864
76	Fetal alcohol syndrome: mild	0.353	0.266~0.449
77	Fetal alcohol syndrome: moderate	0.459	0.364~0.561
78	Fetal alcohol syndrome: severe	0.712	0.624~0.790
79	Cannabis dependence	0.769	0.684~0.840
80	Amphetamine dependence	0.808	0.731~0.870
81	Cocaine dependence	0.738	0.650~0.814
82	Heroin and other opioid dependence	0.814	0.734~0.878
83	Anxiety disorders: mild	0.257	0.180~0.359
84	Anxiety disorders: moderate	0.566	0.469~0.666
85	Anxiety disorders: severe	0.787	0.702~0.856
86	Major depressive disorder: mild episode	0.551	0.453~0.670
87	Major depressive disorder: moderate episode	0.756	0.675~0.844
88	Major depressive disorder: severe episode	0.838	0.762~0.896
89	Bipolar disorder: manic episode	0.658	0.563~0.745
90	Bipolar disorder: residual state	0.248	0.173~0.356
91	Schizophrenia: acute state	0.836	0.762~0.894
92	Schizophrenia, residual state	0.742	0.655~0.822
93	Anorexia nervosa	0.448	0.355~0.548
94	Bulimia nervosa	0.532	0.436~0.626
95	Attention deficit hyperactivity disorder	0.470	0.376~0.566
96	Conduct disorder	0.625	0.533~0.710
97	Asperger's syndrome	0.432	0.337~0.532
98	Autism	0.677	0.586~0.760
99	Intellectual disability: mild	0.493	0.401~0.586
100	Intellectual disability: moderate	0.585	0.485~0.703
101	Intellectual disability: severe	0.652	0.561~0.768
102	Intellectual disability: profound	0.650	0.559~0.755
103	Hearing loss: mild	0.138	0.087~0.206
104	Hearing loss: moderate	0.231	0.160~0.318
105	Hearing loss: severe	0.406	0.314~0.503
106	Hearing loss: profound	0.491	0.395~0.588
107	Hearing loss: complete	0.669	0.577~0.753
108	Hearing loss: mild, with ringing	0.423	0.318~0.533
109	Hearing loss: moderate, with ringing	0.438	0.345~0.535
110	Hearing loss: severe, with ringing	0.449	0.356~0.544
111	Hearing loss: profound, with ringing	0.640	0.537~0.734
112	Hearing loss: complete, with ringing	0.629	0.530~0.734
113	Distance vision: mild impairment	0.084	0.047~0.139
114	Distance vision: moderate impairment	0.475	0.381~0.571
115	Distance vision: severe impairment	0.687	0.592~0.771
116	Distance vision blindness	0.708	0.617~0.789
117	Near vision impairment	0.256	0.181~0.344
118	Low back pain: acute, without leg pain	0.588	0.494~0.677
119	Low back pain: acute, with leg pain	0.595	0.500~0.689
120	Low back pain: chronic, without leg pain	0.524	0.424~0.622
121	Low back pain: chronic, with leg pain	0.554	0.460~0.646
122	Neck pain: acute, mild	0.338	0.254~0.431
123	Neck pain: acute, severe	0.492	0.393~0.592
124	Neck pain: chronic, mild	0.407	0.316~0.503
125	Neck pain: chronic, severe	0.495	0.402~0.595
126	Musculoskeletal problems: legs, mild	0.205	0.138~0.288
127	Musculoskeletal problems: legs, moderate	0.491	0.397~0.585
128	Musculoskeletal problems: legs, severe	0.535	0.441~0.627
129	Musculoskeletal problems: arms, mild	0.344	0.257~0.440
130	Musculoskeletal problems: arms, moderate	0.461	0.366~0.559
131	Musculoskeletal problems: generalised, moderate	0.568	0.474~0.658
132	Musculoskeletal problems: generalised, severe	0.746	0.655~0.828
133	Gout: acute	0.672	0.579~0.756
134	Amputation of finger(s), excluding thumb: long term, with treatment	0.274	0.197~0.363
135	Amputation of thumb: long term	0.272	0.196~0.362
136	Amputation of one arm: long term, with or without treatment	0.634	0.542~0.719
137	Amputation of both arms: long term, with treatment	0.470	0.377~0.565
138	Amputation of both arms: long term, without treatment	0.748	0.660~0.822
139	Amputation of toe	0.252	0.179~0.339
140	Amputation of one leg: long term, with treatment	0.409	0.317~0.505
141	Amputation of one leg: long term, without treatment	0.629	0.536~0.716
142	Amputation of both legs: long term, with treatment	0.492	0.395~0.605
143	Amputation of both legs: long term, without treatment	0.730	0.639~0.808
144	Burns of <20% total surface area without lower airway burns: short term, with or without treatment	0.514	0.420~0.612
145	Burns of <20% total surface area or <10% total surface area if head or neck, or hands or wrist involved: long term, with or without treatment	0.236	0.164~0.322
146	Burns of ≥20% total surface area: short term, with or without treatment	0.514	0.415~0.613
147	Burns of ≥20% total surface area or ≥10% total surface area if head or neck, or hands or wrist involved: long term, with treatment	0.555	0.460~0.647
148	Burns of ≥20% total surface area or ≥10% total surface area if head or neck, or hands or wrist involved: long term, without treatment	0.745	0.656~0.821
149	Lower airway burns: with or without treatment	0.827	0.750~0.888
150	Crush injury: short or long term, with or without treatment	0.325	0.242~0.417
151	Dislocation of hip: long term, with or without treatment	0.311	0.229~0.407
152	Dislocation of knee: long term, with or without treatment	0.598	0.503~0.697
153	Dislocation of shoulder: long term, with or without treatment	0.393	0.291~0.504
154	Other injuries of muscle and tendon (includes sprains, strains and dislocations other than shoulder, knee, or hip)	0.206	0.139~0.288
155	Drowning and non-fatal submersion: short or long term, with or without treatment	0.589	0.495~0.677
156	Fracture of clavicle, scapula, or humerus: short or long term, with or without treatment	0.416	0.325~0.512
157	Fracture of face bone: short or long term, with or without treatment	0.580	0.481~0.674
158	Fracture of foot bones: short term, with or without treatment	0.383	0.293~0.480
159	Fracture of foot bones: long term, without treatment	0.235	0.162~0.323
160	Fracture of hand: short term, with or without treatment	0.238	0.165~0.325
161	Fracture of hand: long term, without treatment	0.117	0.070~0.182
162	Fracture of neck of femur: short term, with or without treatment	0.701	0.592~0.796
163	Fracture of neck of femur: long term, with treatment	0.380	0.291~0.498
164	Fracture of neck of femur: long term, without treatment	0.793	0.711~0.876
165	Fracture, other than neck of femur: short term, with or without treatment	0.702	0.611~0.783
166	Fracture, other than neck of femur: long term, without treatment	0.363	0.277~0.456
167	Fracture of patella, tibia or fibula, or ankle: short term, with or without treatment	0.519	0.422~0.614
168	Fracture of patella, tibia or fibula, or ankle: long term, with or without treatment	0.428	0.335~0.526
169	Fracture of pelvis: short term	0.759	0.671~0.832
170	Fracture of pelvis: long term	0.573	0.479~0.685
171	Fracture of radius or ulna: short term, with or without treatment	0.421	0.326~0.527
172	Fracture of radius or ulna: long term, without treatment	0.473	0.380~0.567
173	Fracture of skull: short or long term, with or without treatment	0.390	0.301~0.485
174	Fracture of sternum or fracture of one or two ribs: short term, with or without treatment	0.500	0.405~0.596
175	Fracture of vertebral column: short or long term, with or without treatment	0.399	0.308~0.496
176	Fractures: treated, long term	0.161	0.103~0.243
177	Injured nerves: short term	0.463	0.369~0.560
178	Injured nerves: long term	0.511	0.415~0.607
179	Injury to eyes: short term	0.409	0.317~0.506
180	Severe traumatic brain injury: short term, with or without treatment	0.479	0.384~0.576
181	Traumatic brain injury: long-term consequences, minor, with or without treatment	0.487	0.392~0.583
182	Traumatic brain injury: long-term consequences, moderate, with or without treatment	0.497	0.403~0.591
183	Traumatic brain injury: long-term consequences, severe, with or without treatment	0.829	0.753~0.888
184	Open wound: short term, with or without treatment	0.208	0.143~0.286
185	Poisoning: short term, with or without treatment	0.400	0.311~0.494
186	Severe chest injury: long term, with or without treatment	0.496	0.399~0.593
187	Severe chest injury: short term, with or without treatment	0.590	0.495~0.681
188	Spinal cord lesion below neck: treated	0.697	0.607~0.776
189	Spinal cord lesion below neck: untreated	0.855	0.782~0.910
190	Spinal cord lesion at neck: treated	0.813	0.736~0.874
191	Spinal cord lesion at neck level: untreated	0.912	0.852~0.952
192	Abdominopelvic problem: mild	0.154	0.098~0.226
193	Abdominopelvic problem: moderate	0.416	0.325~0.512
194	Abdominopelvic problem: severe	0.793	0.711~0.859
195	Anaemia: mild	0.123	0.076~0.188
196	Anaemia: moderate	0.321	0.237~0.415
197	Anaemia: severe	0.453	0.358~0.552
198	Periodontitis	0.095	0.053~0.158
199	Dental caries: symptomatic	0.181	0.117~0.263
200	Severe tooth loss	0.478	0.382~0.576
201	Disfigurement: level 1	0.358	0.271~0.453
202	Disfigurement: level 2	0.605	0.504~0.700
203	Disfigurement: level 3	0.745	0.657~0.821
204	Disfigurement: level 1 with itch or pain	0.419	0.328~0.515
205	Disfigurement: level 2, with itch or pain	0.613	0.511~0.716
206	Disfigurement: level 3, with itch or pain	0.849	0.777~0.905
207	Generic uncomplicated disease: worry and daily medication	0.299	0.221~0.388
208	Generic uncomplicated disease: anxiety about diagnosis	0.411	0.318~0.521
209	Iodine-deficiency goiter	0.548	0.451~0.642
210	Kwashiorkor	0.346	0.262~0.444
211	Severe wasting	0.355	0.269~0.449
212	Speech problems	0.518	0.421~0.614
213	Motor impairment: mild	0.164	0.104~0.267
214	Motor impairment: moderate	0.322	0.237~0.423
215	Motor impairment: severe	0.723	0.633~0.801
216	Motor plus cognitive impairments: mild	0.435	0.341~0.538
217	Motor plus cognitive impairments: moderate	0.509	0.413~0.626
218	Motor plus cognitive impairments: severe	0.790	0.707~0.858
219	Rectovaginal fistula	0.782	0.698~0.850
220	Vesicovaginal fistula	0.686	0.592~0.770
221	Allergic rhinitis & conjunctivitis: mild	0.202	0.135~0.291
222	Allergic rhinitis & conjunctivitis: severe	0.343	0.258~0.437
223	Post-traumatic stress disorder	0.616	0.521~0.706
224	Multiple chemical sensitivity	0.486	0.390~0.589
225	Tinnitus	0.275	0.198~0.364
226	Annoyance: mild	0.169	0.112~0.247
227	annoyance: severe	0.249	0.176~0.335
228	Sleep disorder: mild	0.149	0.093~0.233
229	Sleep disorder: severe	0.328	0.248~0.417
230	Learning disorder: mild	0.157	0.102~0.229
231	Learning disorder: severe	0.156	0.102~0.226
232	EQ-5D 11111	0.116	0.068~0.184
233	EQ-5D 12322	0.404	0.316~0.497
234	EQ-5D 22431	0.355	0.269~0.449
235	EQ-5D 21225	0.619	0.524~0.708
236	EQ-5D 23142	0.514	0.419~0.614
237	EQ-5D 52213	0.562	0.465~0.655
238	EQ-5D 33251	0.458	0.362~0.557
239	EQ-5D 35123	0.576	0.479~0.669
240	EQ-5D 34412	0.516	0.417~0.615
241	EQ-5D 31334	0.643	0.549~0.729
242	EQ-5D 14244	0.673	0.581~0.756
243	EQ-5D 13533	0.403	0.315~0.498
244	EQ-5D 41443	0.591	0.496~0.688
245	EQ-5D 44521	0.615	0.521~0.704
246	EQ-5D 42154	0.683	0.592~0.765
247	EQ-5D 45232	0.641	0.544~0.730
248	EQ-5D 43315	0.670	0.577~0.753
249	EQ-5D 24353	0.609	0.514~0.698
250	EQ-5D 25514	0.718	0.630~0.815
251	EQ-5D 54135	0.719	0.631~0.796
252	EQ-5D 53424	0.765	0.677~0.839
253	EQ-5D 55341	0.558	0.463~0.651
254	EQ-5D 51552	0.744	0.657~0.841
255	EQ-5D 32545	0.708	0.616~0.788
256	EQ-5D 15455	0.772	0.691~0.860

**Fig 2:**
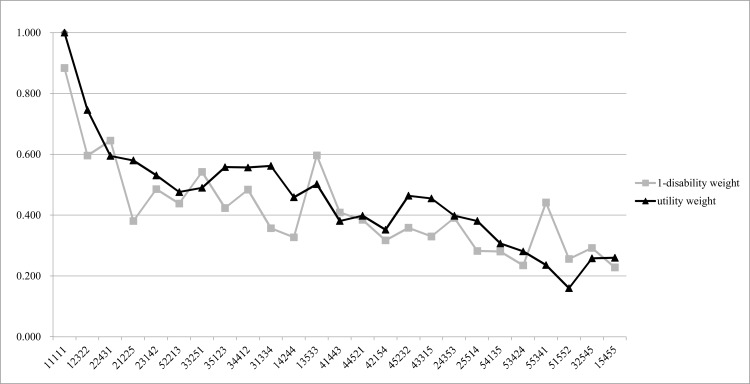
Comparison of the values of 1 minus disability weight and the utility weights from the 25 EQ-5D-5L in Model 1.

Fig 2 shows the results of comparing the values of 1 minus disability weights and the utility weights from the 25 EQ-5D-5L health states in Model 1. The Pearson correlation coefficient between the values of 1 minus disability weights and the utility weights from the 25 EQ-5D-5L health states in Model 1 was 0.8333.

## Discussion

In the present study, the disability weights for the 256 health states were estimated based on the perceptions of the 2,728 participants in the household survey and those of the 3,188 participants in the web-based survey from among the general South Korean population. Four models were used in the analysis of responses and, Model 1, the paired comparison-only model was selected as the best model for estimating the disability weights in South Korea. This is based on the distribution of the disability weights of the health states, the Pearson correlation coefficient, and simplicity of the analysis. Although the Pearson correlation coefficient was highest in Model 2, the difference between the Pearson correlation coefficients from Model 1 and Model 2 was only 0.006 and the discrimination of values according to the severity of the health states was better in Model 1 than in Model 2. We showed that the disability weights could be estimated based only on paired comparison data by including ‘full health’ and ‘being dead’ as anchor points in the compared health state lists.

The results from our current study, and in particular the data generated from Model 1, showed that PHE data are not needed to calculate disability weights. PHE is the revised version of the person trade-off (PTO) provided by Nord [26]. Although there are several variations of PTO analyses [6, 8, 10, 27, 28], the lack of theoretical support, ethical concerns about distributional preference, and the questionable validity of forced consistency have been reported [13, 22, 29]. Nevertheless, in the GBD 2010 disability weight study, the responses to PHE were still used to anchor the results from the paired comparison data on the disability weight scale ranging between 0 and 1 [6]. In the GBD 2013 study, the disability weights were revised by including the results from the European disability weight study, however, PHE data were utilized in addition to PC data to estimate the disability weights [16, 30]. Hence, the use of the trade-off method to link the results from the PHE with paired comparison data was unavoidable. However, the addition of ‘full health’ and ‘being dead’ as anchor points in the process of estimating disability weights could help overcome the concerns of PTO or PHE. In the other valuation method, such as SG and time trade-off, ‘being dead’ is regarded as a reference for eliciting participants’ preferences [2]. In the VAS, the ‘best imaginable health state’ and the ‘worst imaginable health state’ are utilized as references [31]. By adding ‘full health’ and ‘being dead’ to the list of health states, we can the estimate disability weights based on a paired comparison, without relying on PTO or PHE.

Using only paired comparison as a valuation method for estimating disability weight can simplify data analysis. In addition, the analytical methodology for paired comparison data has a sound theoretical basis. For example, Thurstone’s model has been in widespread use for the analysis of paired comparison data since the 1920s [32], and the Bradley-Terry model has also been extensively used [33, 34]. In addition, when survey questions have binary choices, as in the present study, probit regression models such as in Model 1 are appropriate for data analysis [22]. Because paired comparisons are easier for participants to understand and more convenient to employ than PTO or PHE, more consistent responses from participants, in particular, from participants with a low educational level, are obtained [35]. Taken together, these points suggest that the use of a paired comparison-only model is an appropriate method for estimating disability weights in the future.

Some disability weights may appear slightly counterintuitive in terms of the extent and order as compared to others, as the disability weights for numerous health states were estimated to range from 0 to 1. However, it is not easy to assess the validity, particularly the concurrent validity, of disability weights, as there is no gold standard for the disability weights [17]. In the present study, we utilized the EQ-5D-5L health states to evaluate the validity of the disability weights and support the robustness of the analytic methods. When the values of 1 minus disability weights and the utility weights from the 25 EQ-5D-5L health states in Model 1 were compared, there was a fairly high Pearson correlation coefficient between these parameters. In the case of EQ-5D-5L 11111, which indicates no problems in the 5 dimensions, the disability weight was estimated to be 0.116. However, in general, the parameter estimate of constant is included in the models for the EQ-5D-5L valuation study, and the constant variable in the tariff formula for the Korean EQ-5D-5L valuation study (0.096) was found to be similar to the disability weight of EQ-5D-5L 11111 in Model 1 [25]. We cannot determine whether the disability weight or utility weight should be considered as the gold standard; hence, we only compared the utility weights and disability weights from EQ-5D-5L, and did not use them to adjust the results of the analyses.

Another method to confirm the validity of disability weights is to detect the reverse order of disability weights in specific health states with different severity levels (e.g. mild, moderate, and severe) [17]. In Model 1, there was no inversion of disability weights in the health states with different severity levels. For example, in the case of ‘Hearing loss’, the disability weights were 0.138 for mild, 0.231 for moderate, 0.406 for severe, 0.491 for profound, and 0.669 for complete hearing loss. In contrast, the disability weights of ‘Hearing loss’ in the GBD 2010 disability weight study were 0.005 for mild, 0.023 for moderate, 0.032 for severe, 0.031 for profound, and 0.033 for complete hearing loss [6]. In addition, in the GBD 2013 disability weight study, the disability weights of ‘Hearing loss’ were 0.010 for mild, 0.027 for moderate, 0.158 for severe, 0.204 for profound, and 0.215 for complete hearing loss [16]. Thus, the disability weights of ‘Hearing loss’ in the present study were larger than those in the GBD 2010 disability weight study and GBD 2010 disability weight study. Although it is not easy to determine the validity of disability weights, discussions for refining the methodology, including the method of analysis, are needed to determine valid disability weights.

Contextual differences in the perception of health problems are also an important matter. Although the universality of disability weights has been questioned, previous studies have shown conflicting results. A study among western European countries reported a reasonably high level of agreement in the ranking of disability weights [27], and the GBD 2010 disability weight study showed strong evidence of highly consistent results for disability weights between countries [6]. However, another study showed differences in the ranking of the majority of health states between 14 countries, indicating a lack of universality of disability weight assessment [36]. In our current study, comparing the disability weights between Model 1 and the previous GBD 2010 disability weight study, a significantly similar pattern was seen, based on the Pearson correlation coefficient. However, only few reports have investigated the contextual differences in disability weight assessment and further studies are needed to determine the universality of such data. As mentioned above, using a paired comparison-only model may simplify the execution of disability weight studies at the national level. Furthermore, we expect that pooling such data may overcome concerns on the universality of disability weights.

In our study, health state descriptions played an important role in the resultant values of disability weights [6, 17]. We mainly used lay descriptions of health states based on the GBD 2010 disability weight study by translating English into Korean. For this reason, similar patterns of disability weights were seen between our study and the GBD 2010 disability weight study. For example, the disability weights of drug addiction health states were high as compared to the other health states in both studies. We suspect that the social stigma associated with drug addiction may influence the perception of participants, because the lay descriptions of the drug addiction health states included the name of the drugs. This phenomenon became more apparent in the comparisons of the disability weights for health states related to HIV or AIDS, for which the descriptions contained no mention of “HIV” or “AIDS”. We also assumed that this phenomenon might be more prominent in a survey involving the general public than one specifically involving health professionals. Hence, a comparison of the results of a survey of the general public and health professionals would be meaningful, and the re-estimation of the disability weights may be needed to diminish the controversy over these disability weights after modifying the lay description.

One limitation of the present study was that information about the response rate of the household survey and web-based survey was not obtained. Therefore, we could not determine the number of people who refused to participate in both surveys and dropped out during the surveys. Moreover, we could not exclude the possibility of a non-response bias; this limitation may restrict the representativeness of this study in Korea. Another limitation of this study is that we did not verify the responses in the web-based survey. Although the individuals who participated in the web-based survey tended to be younger, those who participated in the household survey tended to have a lesser number of clinical medical problems than the participants in the web-based survey. Hence, quality control, including the verification of responses, will be required in a future web-based survey.

## Conclusions

The paired comparison-only model is the best model for estimating disability weights in South Korea, based on the distribution of the disability weights of the health states, the Pearson correlation coefficient, and the simplicity of the analysis. Hence, disability weights can be estimated using only paired comparisons and by including ‘full health’ and ‘being dead’ as anchor points in the list of health states. Furthermore, we utilized the EQ-5D-5L health states to evaluate the validity of disability weights and determined the robustness of the paired comparison-only model. By adapting and simplifying the methodology of estimating disability weights, as in the present study, we believe that addition empirical evidence on the universality of disability weight can be obtained.

## Supporting Information

S1 File 95% confidence interval in each model. (XLSX)

S1 Table Lay description for added health states. (DOCX)
