# Supplementary material for: Estimation of Disability Weights in the General Population of South Korea Using a Paired Comparison
Source: PLoS One. 2016 Sep 8;11(9):e0162478. doi: 10.1371/journal.pone.0162478 (PMC5015913; doi:10.1371/journal.pone.0162478)
Supplement: S1 Table — (DOCX) [file pone.0162478.s002.docx]

S1 Table. 95% confidence intervals of disability weights in each model.

| N | Health states | Model 1 | | | Model 2 | | | Model 3 | | | Model 4 | | |
| --- | --- | --- | --- | --- | --- | --- | --- | --- | --- | --- | --- | --- | --- |
|  |  | DW | 95% CI | | DW | 95% CI | | DW | 95% CI | | DW | 95% CI | |
|  |  |  | lower | upper |  | lower | upper |  | lower | upper |  | lower | upper |
| 1 | Infectious disease: acute episode, mild | 0.111 | 0.061 | 0.186 | 0.232 | 0.214 | 0.251 | 0.221 | 0.083 | 0.451 | 0.208 | 0.079 | 0.427 |
| 2 | Infectious disease: acute episode, moderate | 0.385 | 0.294 | 0.488 | 0.304 | 0.296 | 0.312 | 0.410 | 0.187 | 0.633 | 0.239 | 0.097 | 0.465 |
| 3 | Infectious disease: acute episode, severe | 0.587 | 0.488 | 0.681 | 0.339 | 0.331 | 0.347 | 0.511 | 0.235 | 0.787 | 0.291 | 0.131 | 0.517 |
| 4 | Infectious disease: post-acute consequences (fatigue, emotional lability, insomnia) | 0.428 | 0.336 | 0.525 | 0.317 | 0.309 | 0.324 | 0.442 | 0.192 | 0.692 | 0.304 | 0.112 | 0.496 |
| 5 | Diarrhoea: mild | 0.359 | 0.271 | 0.462 | 0.299 | 0.291 | 0.308 | 0.438 | 0.188 | 0.687 | 0.300 | 0.012 | 0.588 |
| 6 | Diarrhoea: moderate | 0.535 | 0.438 | 0.630 | 0.320 | 0.312 | 0.327 | 0.455 | 0.224 | 0.686 | 0.278 | 0.123 | 0.504 |
| 7 | Diarrhoea: severe | 0.614 | 0.515 | 0.713 | 0.348 | 0.339 | 0.356 | 0.485 | 0.269 | 0.700 | 0.366 | 0.142 | 0.590 |
| 8 | Epididymo-orchitis | 0.653 | 0.561 | 0.737 | 0.348 | 0.339 | 0.356 | 0.641 | 0.391 | 0.890 | 0.355 | 0.164 | 0.547 |
| 9 | Herpes zoster | 0.323 | 0.240 | 0.415 | 0.284 | 0.273 | 0.294 | 0.446 | 0.171 | 0.720 | 0.110 | 0.021 | 0.343 |
| 10 | HIV cases: symptomatic, pre-AIDS | 0.337 | 0.255 | 0.429 | 0.303 | 0.295 | 0.312 | 0.410 | 0.182 | 0.638 | 0.213 | 0.071 | 0.464 |
| 11 | HIV/AIDS cases: receiving antiretroviral treatment | 0.261 | 0.187 | 0.348 | 0.284 | 0.274 | 0.295 | 0.436 | 0.200 | 0.672 | 0.208 | 0.068 | 0.458 |
| 12 | AIDS cases: not receiving antiretroviral treatment | 0.526 | 0.431 | 0.621 | 0.334 | 0.327 | 0.342 | 0.478 | 0.192 | 0.764 | 0.221 | 0.081 | 0.458 |
| 13 | Intestinal nematode infections: symptomatic | 0.521 | 0.424 | 0.618 | 0.331 | 0.323 | 0.338 | 0.529 | 0.285 | 0.773 | 0.338 | 0.133 | 0.543 |
| 14 | Lymphatic filariasis: symptomatic | 0.473 | 0.382 | 0.565 | 0.316 | 0.308 | 0.323 | 0.458 | 0.200 | 0.716 | 0.234 | 0.098 | 0.449 |
| 15 | Ear pain | 0.241 | 0.169 | 0.327 | 0.273 | 0.261 | 0.285 | 0.353 | 0.025 | 0.681 | 0.202 | 0.079 | 0.410 |
| 16 | Tuberculosis: without HIV infection | 0.537 | 0.439 | 0.652 | 0.333 | 0.326 | 0.341 | 0.422 | 0.173 | 0.670 | 0.255 | 0.099 | 0.499 |
| 17 | Tuberculosis: with HIV infection | 0.461 | 0.366 | 0.560 | 0.319 | 0.311 | 0.326 | 0.414 | 0.201 | 0.627 | 0.361 | 0.138 | 0.585 |
| 18 | Cancer: diagnosis and primary therapy | 0.536 | 0.441 | 0.629 | 0.334 | 0.326 | 0.341 | 0.521 | 0.289 | 0.752 | 0.318 | 0.101 | 0.536 |
| 19 | Cancer: metastatic | 0.596 | 0.495 | 0.691 | 0.346 | 0.337 | 0.354 | 0.607 | 0.396 | 0.819 | 0.242 | 0.088 | 0.493 |
| 20 | Mastectomy | 0.468 | 0.374 | 0.568 | 0.321 | 0.314 | 0.328 | 0.450 | 0.235 | 0.665 | 0.274 | 0.108 | 0.525 |
| 21 | Stoma | 0.634 | 0.542 | 0.721 | 0.359 | 0.349 | 0.369 | 0.668 | 0.459 | 0.877 | 0.414 | 0.121 | 0.708 |
| 22 | Terminal phase: with medication (for cancers, end-stage kidney/liver disease) | 0.733 | 0.642 | 0.811 | 0.376 | 0.364 | 0.388 | 0.637 | 0.307 | 0.966 | 0.443 | 0.219 | 0.668 |
| 23 | Terminal phase, without medication (for cancers, end-stage kidney or liver disease) | 0.737 | 0.649 | 0.813 | 0.370 | 0.358 | 0.381 | 0.648 | 0.420 | 0.876 | 0.461 | 0.203 | 0.719 |
| 24 | Acute myocardial infarction: days 1-2 | 0.575 | 0.480 | 0.682 | 0.338 | 0.331 | 0.346 | 0.553 | 0.278 | 0.828 | 0.231 | 0.073 | 0.507 |
| 25 | Acute myocardial infarction: days 3-28 | 0.390 | 0.300 | 0.486 | 0.303 | 0.295 | 0.311 | 0.430 | 0.181 | 0.679 | 0.250 | 0.112 | 0.456 |
| 26 | Angina pectoris: mild | 0.252 | 0.177 | 0.340 | 0.272 | 0.260 | 0.284 | 0.286 | 0.122 | 0.523 | 0.153 | 0.032 | 0.438 |
| 27 | Angina pectoris: moderate | 0.344 | 0.258 | 0.439 | 0.287 | 0.277 | 0.297 | 0.344 | 0.086 | 0.718 | 0.296 | 0.137 | 0.517 |
| 28 | Angina pectoris: severe | 0.470 | 0.376 | 0.566 | 0.320 | 0.313 | 0.328 | 0.490 | 0.258 | 0.721 | 0.340 | 0.129 | 0.550 |
| 29 | Cardiac conduction disorders and cardiac dysrhythmias | 0.670 | 0.555 | 0.771 | 0.356 | 0.347 | 0.366 | 0.586 | 0.351 | 0.820 | 0.423 | 0.116 | 0.730 |
| 30 | Claudication | 0.320 | 0.236 | 0.413 | 0.287 | 0.277 | 0.297 | 0.399 | 0.080 | 0.717 | 0.304 | 0.099 | 0.509 |
| 31 | Heart failure: mild | 0.305 | 0.226 | 0.394 | 0.281 | 0.270 | 0.292 | 0.340 | 0.120 | 0.561 | 0.156 | 0.039 | 0.411 |
| 32 | Heart failure: moderate | 0.376 | 0.286 | 0.472 | 0.292 | 0.283 | 0.302 | 0.420 | 0.201 | 0.638 | 0.263 | 0.121 | 0.470 |
| 33 | Heart failure: severe | 0.547 | 0.452 | 0.639 | 0.328 | 0.321 | 0.335 | 0.489 | 0.269 | 0.710 | 0.291 | 0.128 | 0.522 |
| 34 | Stroke: long-term consequences, mild | 0.209 | 0.140 | 0.294 | 0.269 | 0.257 | 0.282 | 0.404 | 0.157 | 0.651 | 0.206 | 0.062 | 0.473 |
| 35 | Stroke: long-term consequences, moderate | 0.270 | 0.194 | 0.358 | 0.283 | 0.273 | 0.293 | 0.403 | 0.098 | 0.708 | 0.323 | 0.093 | 0.552 |
| 36 | Stroke: long-term consequences, moderate plus cognition problems | 0.497 | 0.403 | 0.591 | 0.331 | 0.324 | 0.338 | 0.517 | 0.318 | 0.716 | 0.313 | 0.120 | 0.506 |
| 37 | Stroke: long-term consequences, severe | 0.768 | 0.681 | 0.841 | 0.377 | 0.364 | 0.389 | 0.700 | 0.480 | 0.860 | 0.396 | 0.193 | 0.599 |
| 38 | Stroke: long-term consequences, severe plus cognition problems | 0.809 | 0.728 | 0.874 | 0.391 | 0.376 | 0.405 | 0.686 | 0.500 | 0.872 | 0.559 | 0.285 | 0.834 |
| 39 | Diabetic foot | 0.222 | 0.154 | 0.308 | 0.263 | 0.249 | 0.276 | 0.315 | 0.086 | 0.544 | 0.158 | 0.050 | 0.373 |
| 40 | Diabetic neuropathy | 0.628 | 0.531 | 0.719 | 0.347 | 0.339 | 0.356 | 0.452 | 0.202 | 0.702 | 0.376 | 0.175 | 0.577 |
| 41 | Chronic kidney disease (stage IV) | 0.345 | 0.257 | 0.460 | 0.299 | 0.290 | 0.307 | 0.435 | 0.078 | 0.793 | 0.177 | 0.067 | 0.374 |
| 42 | End-stage renal disease: with kidney transplant | 0.200 | 0.135 | 0.279 | 0.255 | 0.240 | 0.269 | 0.361 | 0.157 | 0.565 | 0.122 | 0.021 | 0.392 |
| 43 | End-stage renal disease: on dialysis | 0.713 | 0.626 | 0.816 | 0.365 | 0.355 | 0.376 | 0.583 | 0.301 | 0.866 | 0.307 | 0.030 | 0.585 |
| 44 | Decompensated cirrhosis of the liver | 0.375 | 0.285 | 0.473 | 0.303 | 0.295 | 0.311 | 0.468 | 0.182 | 0.754 | 0.269 | 0.111 | 0.507 |
| 45 | Gastric bleeding | 0.782 | 0.699 | 0.850 | 0.376 | 0.364 | 0.389 | 0.567 | 0.317 | 0.816 | 0.398 | 0.176 | 0.620 |
| 46 | Crohn's disease or ulcerative colitis | 0.620 | 0.526 | 0.707 | 0.348 | 0.339 | 0.356 | 0.519 | 0.275 | 0.763 | 0.342 | 0.095 | 0.589 |
| 47 | Benign prostatic hypertrophy: symptomatic cases | 0.372 | 0.284 | 0.467 | 0.296 | 0.287 | 0.305 | 0.388 | 0.118 | 0.658 | 0.156 | 0.017 | 0.537 |
| 48 | Urinary incontinence | 0.582 | 0.487 | 0.672 | 0.342 | 0.334 | 0.350 | 0.627 | 0.375 | 0.878 | 0.300 | 0.012 | 0.588 |
| 49 | Impotence | 0.450 | 0.358 | 0.546 | 0.309 | 0.301 | 0.316 | 0.509 | 0.250 | 0.767 | 0.378 | 0.140 | 0.615 |
| 50 | Infertility: primary | 0.325 | 0.241 | 0.442 | 0.292 | 0.282 | 0.301 | 0.387 | 0.161 | 0.613 | 0.168 | 0.052 | 0.395 |
| 51 | Infertility: secondary | 0.168 | 0.109 | 0.243 | 0.250 | 0.235 | 0.266 | 0.254 | 0.089 | 0.521 | 0.178 | 0.050 | 0.435 |
| 52 | Asthma: controlled | 0.148 | 0.094 | 0.227 | 0.251 | 0.236 | 0.267 | 0.239 | 0.083 | 0.497 | 0.156 | 0.052 | 0.359 |
| 53 | Asthma: partially controlled | 0.294 | 0.215 | 0.385 | 0.283 | 0.272 | 0.293 | 0.320 | 0.129 | 0.510 | 0.319 | 0.027 | 0.611 |
| 54 | Asthma: uncontrolled | 0.342 | 0.258 | 0.445 | 0.296 | 0.287 | 0.305 | 0.412 | 0.164 | 0.660 | 0.300 | 0.086 | 0.514 |
| 55 | COPD and other chronic respiratory problems: mild | 0.173 | 0.111 | 0.263 | 0.256 | 0.242 | 0.271 | 0.278 | 0.100 | 0.553 | 0.144 | 0.034 | 0.398 |
| 56 | COPD and other chronic respiratory problems: moderate | 0.439 | 0.347 | 0.533 | 0.319 | 0.311 | 0.326 | 0.491 | 0.259 | 0.723 | 0.339 | 0.118 | 0.559 |
| 57 | COPD and other chronic respiratory problems: severe | 0.551 | 0.455 | 0.643 | 0.326 | 0.319 | 0.333 | 0.472 | 0.175 | 0.769 | 0.200 | 0.031 | 0.578 |
| 58 | Dementia: mild | 0.401 | 0.310 | 0.503 | 0.306 | 0.298 | 0.314 | 0.391 | 0.145 | 0.636 | 0.242 | 0.088 | 0.493 |
| 59 | Dementia: moderate | 0.606 | 0.513 | 0.694 | 0.345 | 0.337 | 0.353 | 0.520 | 0.276 | 0.764 | 0.444 | 0.187 | 0.702 |
| 60 | Dementia: severe | 0.804 | 0.723 | 0.868 | 0.385 | 0.371 | 0.398 | 0.715 | 0.494 | 0.870 | 0.463 | 0.187 | 0.738 |
| 61 | Headache: migraine | 0.635 | 0.544 | 0.725 | 0.350 | 0.342 | 0.359 | 0.513 | 0.306 | 0.720 | 0.358 | 0.116 | 0.600 |
| 62 | Headache: tension-type | 0.452 | 0.359 | 0.569 | 0.313 | 0.305 | 0.320 | 0.473 | 0.214 | 0.731 | 0.303 | 0.063 | 0.543 |
| 63 | Multiple sclerosis: mild | 0.428 | 0.335 | 0.527 | 0.314 | 0.306 | 0.321 | 0.457 | 0.207 | 0.707 | 0.402 | 0.185 | 0.619 |
| 64 | Multiple sclerosis: moderate | 0.736 | 0.647 | 0.812 | 0.372 | 0.361 | 0.384 | 0.588 | 0.306 | 0.870 | 0.392 | 0.176 | 0.608 |
| 65 | Multiple sclerosis: severe | 0.801 | 0.720 | 0.872 | 0.379 | 0.367 | 0.392 | 0.661 | 0.428 | 0.893 | 0.377 | 0.162 | 0.592 |
| 66 | Epilepsy: treated, seizure free | 0.449 | 0.355 | 0.586 | 0.315 | 0.308 | 0.323 | 0.423 | 0.226 | 0.620 | 0.450 | 0.154 | 0.746 |
| 67 | Epilepsy: treated, with recent seizures | 0.624 | 0.526 | 0.714 | 0.347 | 0.339 | 0.356 | 0.527 | 0.284 | 0.771 | 0.436 | 0.200 | 0.672 |
| 68 | Epilepsy: untreated | 0.660 | 0.566 | 0.750 | 0.356 | 0.346 | 0.365 | 0.577 | 0.313 | 0.841 | 0.277 | 0.119 | 0.509 |
| 69 | Epilepsy: severe | 0.816 | 0.740 | 0.877 | 0.386 | 0.372 | 0.399 | 0.710 | 0.484 | 0.869 | 0.442 | 0.192 | 0.692 |
| 70 | Parkinson's disease: mild | 0.222 | 0.150 | 0.308 | 0.267 | 0.254 | 0.280 | 0.423 | 0.150 | 0.697 | 0.256 | 0.090 | 0.523 |
| 71 | Parkinson's disease: moderate | 0.474 | 0.378 | 0.573 | 0.325 | 0.318 | 0.333 | 0.491 | 0.247 | 0.735 | 0.335 | 0.134 | 0.535 |
| 72 | Parkinson's disease: severe | 0.742 | 0.655 | 0.816 | 0.369 | 0.357 | 0.380 | 0.561 | 0.182 | 0.941 | 0.528 | 0.312 | 0.744 |
| 73 | Alcohol use disorder: mild | 0.463 | 0.368 | 0.566 | 0.321 | 0.313 | 0.328 | 0.467 | 0.224 | 0.711 | 0.294 | 0.144 | 0.502 |
| 74 | Alcohol use disorder: moderate | 0.612 | 0.515 | 0.702 | 0.341 | 0.333 | 0.349 | 0.489 | 0.222 | 0.756 | 0.304 | 0.104 | 0.504 |
| 75 | Alcohol use disorder: severe | 0.797 | 0.715 | 0.864 | 0.389 | 0.375 | 0.403 | 0.562 | 0.266 | 0.857 | 0.382 | 0.092 | 0.672 |
| 76 | Fetal alcohol syndrome: mild | 0.353 | 0.266 | 0.449 | 0.300 | 0.291 | 0.308 | 0.402 | 0.162 | 0.642 | 0.239 | 0.097 | 0.465 |
| 77 | Fetal alcohol syndrome: moderate | 0.459 | 0.364 | 0.561 | 0.314 | 0.307 | 0.322 | 0.527 | 0.277 | 0.778 | 0.266 | 0.103 | 0.517 |
| 78 | Fetal alcohol syndrome: severe | 0.712 | 0.624 | 0.790 | 0.367 | 0.356 | 0.378 | 0.622 | 0.397 | 0.847 | 0.394 | 0.132 | 0.656 |
| 79 | Cannabis dependence | 0.769 | 0.684 | 0.840 | 0.376 | 0.364 | 0.388 | 0.587 | 0.359 | 0.816 | 0.304 | 0.099 | 0.509 |
| 80 | Amphetamine dependence | 0.808 | 0.731 | 0.870 | 0.382 | 0.369 | 0.395 | 0.569 | 0.313 | 0.826 | 0.370 | 0.133 | 0.607 |
| 81 | Cocaine dependence | 0.738 | 0.650 | 0.814 | 0.375 | 0.363 | 0.387 | 0.595 | 0.332 | 0.858 | 0.356 | 0.148 | 0.564 |
| 82 | Heroin and other opioid dependence | 0.814 | 0.734 | 0.878 | 0.391 | 0.377 | 0.406 | 0.619 | 0.425 | 0.813 | 0.550 | 0.300 | 0.800 |
| 83 | Anxiety disorders: mild | 0.257 | 0.180 | 0.359 | 0.278 | 0.267 | 0.289 | 0.385 | 0.082 | 0.688 | 0.313 | 0.116 | 0.511 |
| 84 | Anxiety disorders: moderate | 0.566 | 0.469 | 0.666 | 0.333 | 0.326 | 0.340 | 0.409 | 0.136 | 0.681 | 0.259 | 0.092 | 0.526 |
| 85 | Anxiety disorders: severe | 0.787 | 0.702 | 0.856 | 0.370 | 0.359 | 0.381 | 0.551 | 0.276 | 0.826 | 0.536 | 0.196 | 0.877 |
| 86 | Major depressive disorder: mild episode | 0.551 | 0.453 | 0.670 | 0.333 | 0.325 | 0.340 | 0.486 | 0.219 | 0.753 | 0.340 | 0.107 | 0.573 |
| 87 | Major depressive disorder: moderate episode | 0.756 | 0.675 | 0.844 | 0.376 | 0.364 | 0.388 | 0.530 | 0.286 | 0.773 | 0.361 | 0.143 | 0.579 |
| 88 | Major depressive disorder: severe episode | 0.838 | 0.762 | 0.896 | 0.391 | 0.377 | 0.406 | 0.672 | 0.463 | 0.880 | 0.563 | 0.288 | 0.837 |
| 89 | Bipolar disorder: manic episode | 0.658 | 0.563 | 0.745 | 0.362 | 0.352 | 0.373 | 0.493 | 0.242 | 0.744 | 0.326 | 0.102 | 0.551 |
| 90 | Bipolar disorder: residual state | 0.248 | 0.173 | 0.356 | 0.282 | 0.271 | 0.293 | 0.387 | 0.161 | 0.613 | 0.225 | 0.081 | 0.469 |
| 91 | Schizophrenia: acute state | 0.836 | 0.762 | 0.894 | 0.388 | 0.374 | 0.402 | 0.584 | 0.320 | 0.847 | 0.483 | 0.276 | 0.690 |
| 92 | Schizophrenia, residual state | 0.742 | 0.655 | 0.822 | 0.377 | 0.365 | 0.390 | 0.548 | 0.263 | 0.834 | 0.294 | 0.109 | 0.568 |
| 93 | Anorexia nervosa | 0.448 | 0.355 | 0.548 | 0.315 | 0.308 | 0.323 | 0.373 | 0.084 | 0.662 | 0.278 | 0.100 | 0.553 |
| 94 | Bulimia nervosa | 0.532 | 0.436 | 0.626 | 0.328 | 0.320 | 0.335 | 0.477 | 0.246 | 0.709 | 0.270 | 0.122 | 0.486 |
| 95 | Attention deficit hyperactivity disorder | 0.470 | 0.376 | 0.566 | 0.309 | 0.301 | 0.317 | 0.373 | 0.129 | 0.617 | 0.381 | 0.128 | 0.633 |
| 96 | Conduct disorder | 0.625 | 0.533 | 0.710 | 0.339 | 0.331 | 0.347 | 0.408 | 0.179 | 0.636 | 0.215 | 0.094 | 0.408 |
| 97 | Asperger's syndrome | 0.432 | 0.337 | 0.532 | 0.317 | 0.310 | 0.325 | 0.444 | 0.159 | 0.729 | 0.406 | 0.193 | 0.619 |
| 98 | Autism | 0.677 | 0.586 | 0.760 | 0.357 | 0.347 | 0.366 | 0.572 | 0.336 | 0.807 | 0.256 | 0.093 | 0.514 |
| 99 | Intellectual disability: mild | 0.493 | 0.401 | 0.586 | 0.331 | 0.323 | 0.338 | 0.536 | 0.325 | 0.747 | 0.347 | 0.156 | 0.537 |
| 100 | Intellectual disability: moderate | 0.585 | 0.485 | 0.703 | 0.340 | 0.332 | 0.348 | 0.578 | 0.369 | 0.788 | 0.355 | 0.120 | 0.590 |
| 101 | Intellectual disability: severe | 0.652 | 0.561 | 0.768 | 0.357 | 0.347 | 0.366 | 0.513 | 0.262 | 0.764 | 0.406 | 0.134 | 0.678 |
| 102 | Intellectual disability: profound | 0.650 | 0.559 | 0.755 | 0.350 | 0.341 | 0.358 | 0.560 | 0.264 | 0.856 | 0.367 | 0.137 | 0.597 |
| 103 | Hearing loss: mild | 0.138 | 0.087 | 0.206 | 0.243 | 0.226 | 0.259 | 0.283 | 0.107 | 0.549 | 0.243 | 0.094 | 0.480 |
| 104 | Hearing loss: moderate | 0.231 | 0.160 | 0.318 | 0.279 | 0.268 | 0.290 | 0.408 | 0.161 | 0.655 | 0.261 | 0.122 | 0.463 |
| 105 | Hearing loss: severe | 0.406 | 0.314 | 0.503 | 0.308 | 0.300 | 0.316 | 0.502 | 0.177 | 0.826 | 0.142 | 0.032 | 0.395 |
| 106 | Hearing loss: profound | 0.491 | 0.395 | 0.588 | 0.325 | 0.318 | 0.332 | 0.520 | 0.289 | 0.752 | 0.370 | 0.151 | 0.589 |
| 107 | Hearing loss: complete | 0.669 | 0.577 | 0.753 | 0.350 | 0.342 | 0.359 | 0.701 | 0.418 | 0.892 | 0.398 | 0.170 | 0.625 |
| 108 | Hearing loss: mild, with ringing | 0.423 | 0.318 | 0.533 | 0.310 | 0.302 | 0.318 | 0.397 | 0.150 | 0.643 | 0.144 | 0.032 | 0.407 |
| 109 | Hearing loss: moderate, with ringing | 0.438 | 0.345 | 0.535 | 0.317 | 0.310 | 0.324 | 0.525 | 0.201 | 0.849 | 0.267 | 0.128 | 0.466 |
| 110 | Hearing loss: severe, with ringing | 0.449 | 0.356 | 0.544 | 0.321 | 0.313 | 0.328 | 0.560 | 0.265 | 0.856 | 0.252 | 0.116 | 0.454 |
| 111 | Hearing loss: profound, with ringing | 0.640 | 0.537 | 0.734 | 0.346 | 0.337 | 0.354 | 0.525 | 0.319 | 0.732 | 0.342 | 0.102 | 0.582 |
| 112 | Hearing loss: complete, with ringing | 0.629 | 0.530 | 0.734 | 0.348 | 0.339 | 0.356 | 0.503 | 0.291 | 0.714 | 0.300 | 0.035 | 0.565 |
| 113 | Distance vision: mild impairment | 0.084 | 0.047 | 0.139 | 0.234 | 0.216 | 0.253 | 0.238 | 0.106 | 0.440 | 0.150 | 0.041 | 0.382 |
| 114 | Distance vision: moderate impairment | 0.475 | 0.381 | 0.571 | 0.315 | 0.307 | 0.322 | 0.445 | 0.187 | 0.702 | 0.367 | 0.089 | 0.644 |
| 115 | Distance vision: severe impairment | 0.687 | 0.592 | 0.771 | 0.364 | 0.354 | 0.375 | 0.609 | 0.388 | 0.831 | 0.358 | 0.146 | 0.571 |
| 116 | Distance vision blindness | 0.708 | 0.617 | 0.789 | 0.371 | 0.360 | 0.383 | 0.647 | 0.444 | 0.850 | 0.464 | 0.227 | 0.701 |
| 117 | Near vision impairment | 0.256 | 0.181 | 0.344 | 0.269 | 0.257 | 0.281 | 0.467 | 0.170 | 0.764 | 0.217 | 0.078 | 0.453 |
| 118 | Low back pain: acute, without leg pain | 0.588 | 0.494 | 0.677 | 0.344 | 0.336 | 0.352 | 0.474 | 0.224 | 0.725 | 0.224 | 0.091 | 0.439 |
| 119 | Low back pain: acute, with leg pain | 0.595 | 0.500 | 0.689 | 0.346 | 0.338 | 0.355 | 0.511 | 0.273 | 0.749 | 0.319 | 0.096 | 0.542 |
| 120 | Low back pain: chronic, without leg pain | 0.524 | 0.424 | 0.622 | 0.328 | 0.320 | 0.335 | 0.492 | 0.225 | 0.759 | 0.354 | 0.159 | 0.549 |
| 121 | Low back pain: chronic, with leg pain | 0.554 | 0.460 | 0.646 | 0.337 | 0.329 | 0.345 | 0.497 | 0.271 | 0.723 | 0.273 | 0.110 | 0.517 |
| 122 | Neck pain: acute, mild | 0.338 | 0.254 | 0.431 | 0.294 | 0.285 | 0.303 | 0.383 | 0.031 | 0.734 | 0.146 | 0.045 | 0.353 |
| 123 | Neck pain: acute, severe | 0.492 | 0.393 | 0.592 | 0.329 | 0.322 | 0.336 | 0.491 | 0.214 | 0.767 | 0.333 | 0.118 | 0.547 |
| 124 | Neck pain: chronic, mild | 0.407 | 0.316 | 0.503 | 0.310 | 0.302 | 0.318 | 0.387 | 0.054 | 0.721 | 0.207 | 0.054 | 0.505 |
| 125 | Neck pain: chronic, severe | 0.495 | 0.402 | 0.595 | 0.326 | 0.319 | 0.334 | 0.390 | 0.110 | 0.670 | 0.253 | 0.097 | 0.497 |
| 126 | Musculoskeletal problems: legs, mild | 0.205 | 0.138 | 0.288 | 0.268 | 0.255 | 0.280 | 0.433 | 0.176 | 0.689 | 0.232 | 0.082 | 0.483 |
| 127 | Musculoskeletal problems: legs, moderate | 0.491 | 0.397 | 0.585 | 0.314 | 0.307 | 0.322 | 0.459 | 0.244 | 0.674 | 0.255 | 0.096 | 0.506 |
| 128 | Musculoskeletal problems: legs, severe | 0.535 | 0.441 | 0.627 | 0.337 | 0.330 | 0.345 | 0.535 | 0.268 | 0.801 | 0.336 | 0.157 | 0.515 |
| 129 | Musculoskeletal problems: arms, mild | 0.344 | 0.257 | 0.440 | 0.289 | 0.279 | 0.299 | 0.346 | 0.020 | 0.673 | 0.271 | 0.125 | 0.482 |
| 130 | Musculoskeletal problems: arms, moderate | 0.461 | 0.366 | 0.559 | 0.320 | 0.313 | 0.328 | 0.446 | 0.196 | 0.696 | 0.265 | 0.080 | 0.573 |
| 131 | Musculoskeletal problems: generalised, moderate | 0.568 | 0.474 | 0.658 | 0.339 | 0.331 | 0.347 | 0.463 | 0.248 | 0.679 | 0.272 | 0.096 | 0.547 |
| 132 | Musculoskeletal problems: generalised, severe | 0.746 | 0.655 | 0.828 | 0.378 | 0.365 | 0.390 | 0.554 | 0.304 | 0.804 | 0.179 | 0.056 | 0.413 |
| 133 | Gout: acute | 0.672 | 0.579 | 0.756 | 0.355 | 0.345 | 0.364 | 0.476 | 0.225 | 0.727 | 0.325 | 0.126 | 0.524 |
| 134 | Amputation of finger(s), excluding thumb: long term, with treatment | 0.274 | 0.197 | 0.363 | 0.292 | 0.283 | 0.302 | 0.382 | 0.102 | 0.661 | 0.265 | 0.096 | 0.531 |
| 135 | Amputation of thumb: long term | 0.272 | 0.196 | 0.362 | 0.281 | 0.270 | 0.292 | 0.356 | 0.081 | 0.632 | 0.129 | 0.039 | 0.319 |
| 136 | Amputation of one arm: long term, with or without treatment | 0.634 | 0.542 | 0.719 | 0.343 | 0.335 | 0.351 | 0.490 | 0.193 | 0.788 | 0.389 | 0.162 | 0.615 |
| 137 | Amputation of both arms: long term, with treatment | 0.470 | 0.377 | 0.565 | 0.321 | 0.313 | 0.328 | 0.462 | 0.176 | 0.748 | 0.245 | 0.093 | 0.489 |
| 138 | Amputation of both arms: long term, without treatment | 0.748 | 0.660 | 0.822 | 0.367 | 0.356 | 0.378 | 0.576 | 0.379 | 0.773 | 0.459 | 0.119 | 0.799 |
| 139 | Amputation of toe | 0.252 | 0.179 | 0.339 | 0.279 | 0.268 | 0.290 | 0.389 | 0.187 | 0.592 | 0.213 | 0.080 | 0.438 |
| 140 | Amputation of one leg: long term, with treatment | 0.409 | 0.317 | 0.505 | 0.313 | 0.306 | 0.321 | 0.477 | 0.257 | 0.698 | 0.150 | 0.032 | 0.423 |
| 141 | Amputation of one leg: long term, without treatment | 0.629 | 0.536 | 0.716 | 0.345 | 0.337 | 0.353 | 0.505 | 0.253 | 0.756 | 0.393 | 0.166 | 0.620 |
| 142 | Amputation of both legs: long term, with treatment | 0.492 | 0.395 | 0.605 | 0.321 | 0.314 | 0.329 | 0.451 | 0.263 | 0.639 | 0.387 | 0.200 | 0.575 |
| 143 | Amputation of both legs: long term, without treatment | 0.730 | 0.639 | 0.808 | 0.372 | 0.361 | 0.384 | 0.613 | 0.411 | 0.815 | 0.469 | 0.232 | 0.706 |
| 144 | Burns of <20% total surface area without lower airway burns: short term, with or without treatment | 0.514 | 0.420 | 0.612 | 0.320 | 0.313 | 0.327 | 0.474 | 0.237 | 0.711 | 0.337 | 0.122 | 0.552 |
| 145 | Burns of <20% total surface area or <10% total surface area if head or neck, or hands or wrist involved: long term, with or without treatment | 0.236 | 0.164 | 0.322 | 0.279 | 0.267 | 0.290 | 0.387 | 0.176 | 0.598 | 0.191 | 0.065 | 0.420 |
| 146 | Burns of ≥20% total surface area: short term, with or without treatment | 0.514 | 0.415 | 0.613 | 0.328 | 0.321 | 0.336 | 0.518 | 0.297 | 0.739 | 0.335 | 0.149 | 0.521 |
| 147 | Burns of ≥20% total surface area or ≥10% total surface area if head or neck, or hands or wrist involved: long term, with treatment | 0.555 | 0.460 | 0.647 | 0.338 | 0.330 | 0.346 | 0.489 | 0.263 | 0.715 | 0.433 | 0.198 | 0.669 |
| 148 | Burns of ≥20% total surface area or ≥10% total surface area if head or neck, or hands or wrist involved: long term, without treatment | 0.745 | 0.656 | 0.821 | 0.372 | 0.361 | 0.384 | 0.577 | 0.282 | 0.872 | 0.448 | 0.238 | 0.658 |
| 149 | Lower airway burns: with or without treatment | 0.827 | 0.750 | 0.888 | 0.392 | 0.378 | 0.407 | 0.588 | 0.340 | 0.835 | 0.341 | 0.120 | 0.562 |
| 150 | Crush injury: short or long term, with or without treatment | 0.325 | 0.242 | 0.417 | 0.299 | 0.290 | 0.307 | 0.418 | 0.177 | 0.660 | 0.276 | 0.126 | 0.492 |
| 151 | Dislocation of hip: long term, with or without treatment | 0.311 | 0.229 | 0.407 | 0.284 | 0.274 | 0.295 | 0.380 | 0.193 | 0.568 | 0.100 | 0.019 | 0.324 |
| 152 | Dislocation of knee: long term, with or without treatment | 0.598 | 0.503 | 0.697 | 0.342 | 0.334 | 0.349 | 0.489 | 0.222 | 0.756 | 0.328 | 0.067 | 0.589 |
| 153 | Dislocation of shoulder: long term, with or without treatment | 0.393 | 0.291 | 0.504 | 0.308 | 0.300 | 0.316 | 0.441 | 0.175 | 0.706 | 0.283 | 0.111 | 0.541 |
| 154 | Other injuries of muscle and tendon (includes sprains, strains and dislocations other than shoulder, knee, or hip) | 0.206 | 0.139 | 0.288 | 0.264 | 0.251 | 0.277 | 0.286 | 0.091 | 0.592 | 0.265 | 0.064 | 0.616 |
| 155 | Drowning and non-fatal submersion: short or long term, with or without treatment | 0.589 | 0.495 | 0.677 | 0.332 | 0.325 | 0.340 | 0.490 | 0.214 | 0.767 | 0.257 | 0.103 | 0.495 |
| 156 | Fracture of clavicle, scapula, or humerus: short or long term, with or without treatment | 0.416 | 0.325 | 0.512 | 0.314 | 0.306 | 0.321 | 0.482 | 0.238 | 0.726 | 0.274 | 0.101 | 0.540 |
| 157 | Fracture of face bone: short or long term, with or without treatment | 0.580 | 0.481 | 0.674 | 0.331 | 0.324 | 0.338 | 0.392 | 0.159 | 0.624 | 0.263 | 0.101 | 0.514 |
| 158 | Fracture of foot bones: short term, with or without treatment | 0.383 | 0.293 | 0.480 | 0.293 | 0.284 | 0.302 | 0.368 | 0.149 | 0.586 | 0.242 | 0.103 | 0.457 |
| 159 | Fracture of foot bones: long term, without treatment | 0.235 | 0.162 | 0.323 | 0.279 | 0.268 | 0.290 | 0.387 | 0.172 | 0.603 | 0.350 | 0.133 | 0.567 |
| 160 | Fracture of hand: short term, with or without treatment | 0.238 | 0.165 | 0.325 | 0.269 | 0.256 | 0.281 | 0.380 | 0.136 | 0.625 | 0.173 | 0.051 | 0.413 |
| 161 | Fracture of hand: long term, without treatment | 0.117 | 0.070 | 0.182 | 0.247 | 0.231 | 0.263 | 0.349 | 0.074 | 0.623 | 0.219 | 0.090 | 0.429 |
| 162 | Fracture of neck of femur: short term, with or without treatment | 0.701 | 0.592 | 0.796 | 0.358 | 0.348 | 0.368 | 0.609 | 0.382 | 0.835 | 0.257 | 0.108 | 0.482 |
| 163 | Fracture of neck of femur: long term, with treatment | 0.380 | 0.291 | 0.498 | 0.301 | 0.293 | 0.309 | 0.385 | 0.159 | 0.611 | 0.436 | 0.206 | 0.666 |
| 164 | Fracture of neck of femur: long term, without treatment | 0.793 | 0.711 | 0.876 | 0.383 | 0.369 | 0.396 | 0.612 | 0.373 | 0.850 | 0.502 | 0.271 | 0.734 |
| 165 | Fracture, other than neck of femur: short term, with or without treatment | 0.702 | 0.611 | 0.783 | 0.357 | 0.348 | 0.367 | 0.540 | 0.325 | 0.756 | 0.380 | 0.101 | 0.659 |
| 166 | Fracture, other than neck of femur: long term, without treatment | 0.363 | 0.277 | 0.456 | 0.303 | 0.295 | 0.311 | 0.472 | 0.186 | 0.758 | 0.260 | 0.113 | 0.481 |
| 167 | Fracture of patella, tibia or fibula, or ankle: short term, with or without treatment | 0.519 | 0.422 | 0.614 | 0.322 | 0.315 | 0.329 | 0.446 | 0.215 | 0.676 | 0.271 | 0.088 | 0.567 |
| 168 | Fracture of patella, tibia or fibula, or ankle: long term, with or without treatment | 0.428 | 0.335 | 0.526 | 0.308 | 0.300 | 0.316 | 0.397 | 0.105 | 0.689 | 0.250 | 0.096 | 0.494 |
| 169 | Fracture of pelvis: short term | 0.759 | 0.671 | 0.832 | 0.370 | 0.358 | 0.381 | 0.558 | 0.274 | 0.843 | 0.319 | 0.076 | 0.563 |
| 170 | Fracture of pelvis: long term | 0.573 | 0.479 | 0.685 | 0.338 | 0.331 | 0.346 | 0.562 | 0.267 | 0.858 | 0.261 | 0.082 | 0.557 |
| 171 | Fracture of radius or ulna: short term, with or without treatment | 0.421 | 0.326 | 0.527 | 0.310 | 0.302 | 0.317 | 0.434 | 0.220 | 0.648 | 0.281 | 0.138 | 0.480 |
| 172 | Fracture of radius or ulna: long term, without treatment | 0.473 | 0.380 | 0.567 | 0.310 | 0.302 | 0.318 | 0.416 | 0.193 | 0.639 | 0.193 | 0.062 | 0.435 |
| 173 | Fracture of skull: short or long term, with or without treatment | 0.390 | 0.301 | 0.485 | 0.305 | 0.297 | 0.313 | 0.425 | 0.103 | 0.746 | 0.276 | 0.121 | 0.502 |
| 174 | Fracture of sternum or fracture of one or two ribs: short term, with or without treatment | 0.500 | 0.405 | 0.596 | 0.330 | 0.323 | 0.338 | 0.485 | 0.199 | 0.771 | 0.353 | 0.111 | 0.594 |
| 175 | Fracture of vertebral column: short or long term, with or without treatment | 0.399 | 0.308 | 0.496 | 0.312 | 0.305 | 0.320 | 0.495 | 0.251 | 0.739 | 0.252 | 0.103 | 0.484 |
| 176 | Fractures: treated, long term | 0.161 | 0.103 | 0.243 | 0.247 | 0.231 | 0.263 | 0.360 | 0.131 | 0.589 | 0.235 | 0.078 | 0.502 |
| 177 | Injured nerves: short term | 0.463 | 0.369 | 0.560 | 0.321 | 0.314 | 0.329 | 0.515 | 0.283 | 0.747 | 0.329 | 0.129 | 0.529 |
| 178 | Injured nerves: long term | 0.511 | 0.415 | 0.607 | 0.317 | 0.310 | 0.325 | 0.442 | 0.185 | 0.699 | 0.379 | 0.134 | 0.623 |
| 179 | Injury to eyes: short term | 0.409 | 0.317 | 0.506 | 0.310 | 0.303 | 0.318 | 0.459 | 0.228 | 0.690 | 0.215 | 0.075 | 0.458 |
| 180 | Severe traumatic brain injury: short term, with or without treatment | 0.479 | 0.384 | 0.576 | 0.325 | 0.318 | 0.333 | 0.422 | 0.193 | 0.651 | 0.267 | 0.109 | 0.504 |
| 181 | Traumatic brain injury: long-term consequences, minor, with or without treatment | 0.487 | 0.392 | 0.583 | 0.314 | 0.307 | 0.322 | 0.425 | 0.160 | 0.689 | 0.293 | 0.133 | 0.519 |
| 182 | Traumatic brain injury: long-term consequences, moderate, with or without treatment | 0.497 | 0.403 | 0.591 | 0.332 | 0.325 | 0.340 | 0.521 | 0.270 | 0.772 | 0.284 | 0.124 | 0.516 |
| 183 | Traumatic brain injury: long-term consequences, severe, with or without treatment | 0.829 | 0.753 | 0.888 | 0.392 | 0.377 | 0.406 | 0.592 | 0.299 | 0.885 | 0.544 | 0.334 | 0.755 |
| 184 | Open wound: short term, with or without treatment | 0.208 | 0.143 | 0.286 | 0.268 | 0.255 | 0.280 | 0.305 | 0.084 | 0.525 | 0.270 | 0.115 | 0.502 |
| 185 | Poisoning: short term, with or without treatment | 0.400 | 0.311 | 0.494 | 0.301 | 0.292 | 0.309 | 0.394 | 0.113 | 0.675 | 0.241 | 0.098 | 0.467 |
| 186 | Severe chest injury: long term, with or without treatment | 0.496 | 0.399 | 0.593 | 0.315 | 0.308 | 0.322 | 0.406 | 0.172 | 0.640 | 0.155 | 0.040 | 0.402 |
| 187 | Severe chest injury: short term, with or without treatment | 0.590 | 0.495 | 0.681 | 0.344 | 0.335 | 0.352 | 0.453 | 0.222 | 0.683 | 0.222 | 0.090 | 0.436 |
| 188 | Spinal cord lesion below neck: treated | 0.697 | 0.607 | 0.776 | 0.354 | 0.345 | 0.363 | 0.575 | 0.319 | 0.831 | 0.374 | 0.130 | 0.618 |
| 189 | Spinal cord lesion below neck: untreated | 0.855 | 0.782 | 0.910 | 0.401 | 0.385 | 0.417 | 0.764 | 0.455 | 0.936 | 0.398 | 0.191 | 0.605 |
| 190 | Spinal cord lesion at neck: treated | 0.813 | 0.736 | 0.874 | 0.391 | 0.376 | 0.405 | 0.808 | 0.579 | 0.934 | 0.676 | 0.452 | 0.900 |
| 191 | Spinal cord lesion at neck level: untreated | 0.912 | 0.852 | 0.952 | 0.421 | 0.402 | 0.441 | 0.696 | 0.407 | 0.984 | 0.575 | 0.219 | 0.931 |
| 192 | Abdominopelvic problem: mild | 0.154 | 0.098 | 0.226 | 0.242 | 0.226 | 0.259 | 0.294 | 0.124 | 0.537 | 0.252 | 0.103 | 0.484 |
| 193 | Abdominopelvic problem: moderate | 0.416 | 0.325 | 0.512 | 0.313 | 0.306 | 0.321 | 0.492 | 0.276 | 0.708 | 0.143 | 0.041 | 0.362 |
| 194 | Abdominopelvic problem: severe | 0.793 | 0.711 | 0.859 | 0.383 | 0.370 | 0.396 | 0.581 | 0.376 | 0.785 | 0.422 | 0.217 | 0.627 |
| 195 | Anaemia: mild | 0.123 | 0.076 | 0.188 | 0.236 | 0.218 | 0.254 | 0.250 | 0.106 | 0.470 | 0.119 | 0.037 | 0.292 |
| 196 | Anaemia: moderate | 0.321 | 0.237 | 0.415 | 0.295 | 0.286 | 0.304 | 0.374 | 0.159 | 0.588 | 0.227 | 0.091 | 0.447 |
| 197 | Anaemia: severe | 0.453 | 0.358 | 0.552 | 0.325 | 0.318 | 0.333 | 0.418 | 0.183 | 0.653 | 0.300 | 0.053 | 0.547 |
| 198 | Periodontitis | 0.095 | 0.053 | 0.158 | 0.240 | 0.223 | 0.257 | 0.213 | 0.060 | 0.500 | 0.353 | 0.118 | 0.587 |
| 199 | Dental caries: symptomatic | 0.181 | 0.117 | 0.263 | 0.260 | 0.246 | 0.274 | 0.263 | 0.083 | 0.559 | 0.142 | 0.032 | 0.395 |
| 200 | Severe tooth loss | 0.478 | 0.382 | 0.576 | 0.320 | 0.313 | 0.328 | 0.523 | 0.285 | 0.760 | 0.348 | 0.137 | 0.559 |
| 201 | Disfigurement: level 1 | 0.358 | 0.271 | 0.453 | 0.301 | 0.292 | 0.309 | 0.489 | 0.237 | 0.740 | 0.320 | 0.107 | 0.532 |
| 202 | Disfigurement: level 2 | 0.605 | 0.504 | 0.700 | 0.343 | 0.335 | 0.351 | 0.515 | 0.289 | 0.741 | 0.374 | 0.143 | 0.605 |
| 203 | Disfigurement: level 3 | 0.745 | 0.657 | 0.821 | 0.378 | 0.365 | 0.390 | 0.569 | 0.247 | 0.891 | 0.507 | 0.304 | 0.710 |
| 204 | Disfigurement: level 1 with itch or pain | 0.419 | 0.328 | 0.515 | 0.310 | 0.302 | 0.317 | 0.394 | 0.167 | 0.621 | 0.281 | 0.109 | 0.539 |
| 205 | Disfigurement: level 2, with itch or pain | 0.613 | 0.511 | 0.716 | 0.345 | 0.337 | 0.354 | 0.495 | 0.263 | 0.727 | 0.361 | 0.138 | 0.585 |
| 206 | Disfigurement: level 3, with itch or pain | 0.849 | 0.777 | 0.905 | 0.403 | 0.387 | 0.419 | 0.682 | 0.377 | 0.987 | 0.425 | 0.196 | 0.654 |
| 207 | Generic uncomplicated disease: worry and daily medication | 0.299 | 0.221 | 0.388 | 0.284 | 0.274 | 0.294 | 0.433 | 0.197 | 0.669 | 0.154 | 0.047 | 0.374 |
| 208 | Generic uncomplicated disease: anxiety about diagnosis | 0.411 | 0.318 | 0.521 | 0.306 | 0.299 | 0.314 | 0.407 | 0.189 | 0.624 | 0.369 | 0.139 | 0.599 |
| 209 | Iodine-deficiency goiter | 0.548 | 0.451 | 0.642 | 0.332 | 0.324 | 0.339 | 0.497 | 0.230 | 0.764 | 0.302 | 0.088 | 0.517 |
| 210 | Kwashiorkor | 0.346 | 0.262 | 0.444 | 0.291 | 0.281 | 0.300 | 0.410 | 0.187 | 0.632 | 0.204 | 0.082 | 0.408 |
| 211 | Severe wasting | 0.355 | 0.269 | 0.449 | 0.291 | 0.282 | 0.301 | 0.439 | 0.241 | 0.636 | 0.353 | 0.111 | 0.594 |
| 212 | Speech problems | 0.518 | 0.421 | 0.614 | 0.320 | 0.312 | 0.327 | 0.447 | 0.150 | 0.743 | 0.196 | 0.074 | 0.409 |
| 213 | Motor impairment: mild | 0.164 | 0.104 | 0.267 | 0.258 | 0.243 | 0.272 | 0.344 | 0.110 | 0.577 | 0.165 | 0.054 | 0.380 |
| 214 | Motor impairment: moderate | 0.322 | 0.237 | 0.423 | 0.295 | 0.286 | 0.304 | 0.416 | 0.175 | 0.657 | 0.183 | 0.045 | 0.469 |
| 215 | Motor impairment: severe | 0.723 | 0.633 | 0.801 | 0.367 | 0.356 | 0.377 | 0.622 | 0.421 | 0.823 | 0.404 | 0.204 | 0.603 |
| 216 | Motor plus cognitive impairments: mild | 0.435 | 0.341 | 0.538 | 0.312 | 0.304 | 0.319 | 0.421 | 0.186 | 0.656 | 0.292 | 0.120 | 0.542 |
| 217 | Motor plus cognitive impairments: moderate | 0.509 | 0.413 | 0.626 | 0.326 | 0.319 | 0.334 | 0.519 | 0.308 | 0.730 | 0.294 | 0.144 | 0.502 |
| 218 | Motor plus cognitive impairments: severe | 0.790 | 0.707 | 0.858 | 0.378 | 0.366 | 0.391 | 0.623 | 0.371 | 0.874 | 0.444 | 0.229 | 0.659 |
| 219 | Rectovaginal fistula | 0.782 | 0.698 | 0.850 | 0.375 | 0.363 | 0.386 | 0.586 | 0.279 | 0.892 | 0.386 | 0.160 | 0.613 |
| 220 | Vesicovaginal fistula | 0.686 | 0.592 | 0.770 | 0.364 | 0.353 | 0.374 | 0.685 | 0.426 | 0.944 | 0.350 | 0.094 | 0.606 |
| 221 | Allergic rhinitis & conjunctivitis: mild | 0.202 | 0.135 | 0.291 | 0.265 | 0.252 | 0.278 | 0.295 | 0.134 | 0.521 | 0.238 | 0.108 | 0.436 |
| 222 | Allergic rhinitis & conjunctivitis: severe | 0.343 | 0.258 | 0.437 | 0.293 | 0.284 | 0.302 | 0.398 | 0.199 | 0.597 | 0.205 | 0.086 | 0.401 |
| 223 | Post-traumatic stress disorder | 0.616 | 0.521 | 0.706 | 0.346 | 0.337 | 0.354 | 0.430 | 0.108 | 0.751 | 0.300 | 0.044 | 0.556 |
| 224 | Multiple chemical sensitivity | 0.486 | 0.390 | 0.589 | 0.316 | 0.309 | 0.324 | 0.459 | 0.228 | 0.690 | 0.308 | 0.103 | 0.514 |
| 225 | Tinnitus | 0.275 | 0.198 | 0.364 | 0.277 | 0.265 | 0.288 | 0.429 | 0.224 | 0.634 | 0.238 | 0.076 | 0.514 |
| 226 | Annoyance: mild | 0.169 | 0.112 | 0.247 | 0.253 | 0.239 | 0.268 | 0.273 | 0.113 | 0.510 | 0.278 | 0.132 | 0.485 |
| 227 | annoyance: severe | 0.249 | 0.176 | 0.335 | 0.269 | 0.257 | 0.282 | 0.326 | 0.117 | 0.534 | 0.205 | 0.071 | 0.441 |
| 228 | Sleep disorder: mild | 0.149 | 0.093 | 0.233 | 0.248 | 0.232 | 0.264 | 0.280 | 0.083 | 0.599 | 0.243 | 0.109 | 0.445 |
| 229 | Sleep disorder: severe | 0.328 | 0.248 | 0.417 | 0.287 | 0.278 | 0.297 | 0.428 | 0.172 | 0.685 | 0.273 | 0.084 | 0.580 |
| 230 | Learning disorder: mild | 0.157 | 0.102 | 0.229 | 0.239 | 0.222 | 0.256 | 0.309 | 0.112 | 0.506 | 0.152 | 0.049 | 0.355 |
| 231 | Learning disorder: severe | 0.156 | 0.102 | 0.226 | 0.254 | 0.239 | 0.269 | 0.384 | 0.139 | 0.629 | 0.210 | 0.089 | 0.406 |
| 232 | EQ-5D 11111 | 0.116 | 0.068 | 0.184 | 0.238 | 0.220 | 0.255 | 0.162 | 0.051 | 0.382 | 0.096 | 0.019 | 0.306 |
| 233 | EQ-5D 12322 | 0.404 | 0.316 | 0.497 | 0.300 | 0.292 | 0.308 | 0.410 | 0.163 | 0.658 | 0.175 | 0.052 | 0.416 |
| 234 | EQ-5D 22431 | 0.355 | 0.269 | 0.449 | 0.304 | 0.296 | 0.312 | 0.463 | 0.178 | 0.749 | 0.306 | 0.101 | 0.511 |
| 235 | EQ-5D 21225 | 0.619 | 0.524 | 0.708 | 0.339 | 0.331 | 0.347 | 0.521 | 0.301 | 0.742 | 0.278 | 0.136 | 0.477 |
| 236 | EQ-5D 23142 | 0.514 | 0.419 | 0.614 | 0.323 | 0.316 | 0.330 | 0.515 | 0.218 | 0.813 | 0.231 | 0.094 | 0.451 |
| 237 | EQ-5D 52213 | 0.562 | 0.465 | 0.655 | 0.334 | 0.327 | 0.342 | 0.515 | 0.277 | 0.753 | 0.333 | 0.088 | 0.579 |
| 238 | EQ-5D 33251 | 0.458 | 0.362 | 0.557 | 0.318 | 0.311 | 0.326 | 0.480 | 0.221 | 0.739 | 0.235 | 0.078 | 0.502 |
| 239 | EQ-5D 35123 | 0.576 | 0.479 | 0.669 | 0.332 | 0.324 | 0.339 | 0.475 | 0.292 | 0.657 | 0.270 | 0.117 | 0.496 |
| 240 | EQ-5D 34412 | 0.516 | 0.417 | 0.615 | 0.331 | 0.324 | 0.338 | 0.534 | 0.276 | 0.792 | 0.320 | 0.117 | 0.523 |
| 241 | EQ-5D 31334 | 0.643 | 0.549 | 0.729 | 0.344 | 0.335 | 0.352 | 0.477 | 0.211 | 0.744 | 0.225 | 0.055 | 0.548 |
| 242 | EQ-5D 14244 | 0.673 | 0.581 | 0.756 | 0.350 | 0.342 | 0.359 | 0.560 | 0.350 | 0.770 | 0.306 | 0.057 | 0.554 |
| 243 | EQ-5D 13533 | 0.403 | 0.315 | 0.498 | 0.313 | 0.305 | 0.320 | 0.501 | 0.234 | 0.768 | 0.326 | 0.122 | 0.530 |
| 244 | EQ-5D 41443 | 0.591 | 0.496 | 0.688 | 0.343 | 0.335 | 0.351 | 0.563 | 0.298 | 0.828 | 0.344 | 0.147 | 0.542 |
| 245 | EQ-5D 44521 | 0.615 | 0.521 | 0.704 | 0.346 | 0.338 | 0.355 | 0.560 | 0.317 | 0.802 | 0.271 | 0.099 | 0.537 |
| 246 | EQ-5D 42154 | 0.683 | 0.592 | 0.765 | 0.362 | 0.351 | 0.372 | 0.534 | 0.297 | 0.771 | 0.409 | 0.181 | 0.637 |
| 247 | EQ-5D 45232 | 0.641 | 0.544 | 0.730 | 0.353 | 0.344 | 0.362 | 0.532 | 0.256 | 0.808 | 0.263 | 0.101 | 0.514 |
| 248 | EQ-5D 43315 | 0.670 | 0.577 | 0.753 | 0.349 | 0.341 | 0.358 | 0.593 | 0.321 | 0.865 | 0.279 | 0.117 | 0.516 |
| 249 | EQ-5D 24353 | 0.609 | 0.514 | 0.698 | 0.341 | 0.333 | 0.349 | 0.535 | 0.340 | 0.730 | 0.320 | 0.107 | 0.532 |
| 250 | EQ-5D 25514 | 0.718 | 0.630 | 0.815 | 0.364 | 0.353 | 0.374 | 0.549 | 0.274 | 0.824 | 0.360 | 0.125 | 0.595 |
| 251 | EQ-5D 54135 | 0.719 | 0.631 | 0.796 | 0.362 | 0.351 | 0.372 | 0.566 | 0.292 | 0.840 | 0.309 | 0.093 | 0.525 |
| 252 | EQ-5D 53424 | 0.765 | 0.677 | 0.839 | 0.380 | 0.367 | 0.393 | 0.621 | 0.383 | 0.859 | 0.297 | 0.156 | 0.486 |
| 253 | EQ-5D 55341 | 0.558 | 0.463 | 0.651 | 0.346 | 0.338 | 0.354 | 0.505 | 0.273 | 0.737 | 0.434 | 0.185 | 0.683 |
| 254 | EQ-5D 51552 | 0.744 | 0.657 | 0.841 | 0.369 | 0.358 | 0.380 | 0.633 | 0.429 | 0.838 | 0.315 | 0.104 | 0.527 |
| 255 | EQ-5D 32545 | 0.708 | 0.616 | 0.788 | 0.359 | 0.350 | 0.369 | 0.489 | 0.245 | 0.733 | 0.362 | 0.170 | 0.554 |
| 256 | EQ-5D 15455 | 0.772 | 0.691 | 0.860 | 0.375 | 0.363 | 0.387 | 0.580 | 0.345 | 0.814 | 0.367 | 0.052 | 0.681 |

DW, disability weight; CI, confidence interval
